# Supplementary material for: Development of a T Cell-Based COVID-19 Vaccine Using a Live Attenuated Influenza Vaccine Viral Vector
Source: Vaccines (Basel). 2022 Jul 18;10(7):1142. doi: 10.3390/vaccines10071142 (PMC9318028; doi:10.3390/vaccines10071142)
Supplement: Supplementary file 1 [file vaccines-10-01142-s001.zip › vaccines-1775091-supplementary.pdf]

Supplementary Materials

Table S1. A list of HLA-A*02:01 restricted peptides used to assess T-cell immune responses to SARS-CoV-2 virus in HLA-A2.1 transgenic mice

№	Peptide	IEDB ID	Position on protein
Peptide mix #1			
1	RLNEVAKNL	54680	S (1185-1193)
2	NLNESLIDL	44814	S (1192-1200)
3	GMSRIGMEV	21347	N (316-324)
4	LALLLLDRL	34851	N (219-227)
5	LLLDRLNQL	37473	N (222-230)
6	FIAGLIAIV	16156	S (1220-1228)
7	TLACFVLAAV	64710	M (61-70)
8	VVFLHVTYV	71663	S (1060-1068)
Peptide mix #2			
9	FLWLLWPVT	16972	M (53-61)
10	FVLAAVYRI	18219	M (65-73)
11	AQFAPSASA	3956	N (305-313)
12	ALNTPKDHI	2802	N (138-146)
13	LQLPQGTTL	38881	N (159-167)
14	ILLNKHIDA	27182	N (351-359)
15	ALNTLVKQL	2801	S (958-966)
16	VLNDILSRL	69657	S (976-984)
17	LITGRLQSL	36724	S (996-1004)

Table S2. Demographic characteristics of COVID-19 patients who donated whole blood for this study

# of PBMC specimen	Sex	Age, years	Disease severity	Time post symptoms onset
1	M	31	mild	4 months
2	M	26	moderate	3 months
3	F	28	mild	3 months
4	M	31	mild	2 months
5	F	46	severe	2 months
6	F	25	mild	2 months
7	F	41	severe	3 months
8	F	39	moderate	3 months
9	M	39	mild	1 months
10	F	47	severe	2 months
11	M	34	mild	3 months
12	F	30	mild	3 months
13	M	30	mild	3 months
14	F	31	mild	5 months
15	F	45	mild	1 months
16	M	47	mild	1 months
17	F	18	mild	1 months
18	F	39	moderate	2 months
19	F	30	mild	2 months
20	M	30	mild	2 months

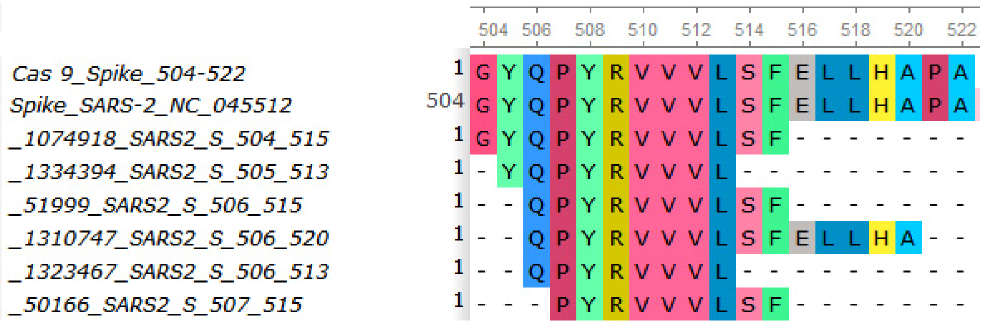

Figure S1. 504-522 fragment of spike protein aligned with peptides established as immunogenic/MHC molecule binding epitopes in experimental studies. This fragment is a part of cassette #9. Epitopes' IDs in Immune Epitope Database are indicated on the left. References and additional information regarding these epitopes are listed in Table S3. This fragment of spike protein was predicted to be involved in interaction with ACE receptor [1].

Table S3. Epitopes in 504-522 fragment of spike protein, deposited in IEDB, additional information and references

Epitope ID	Epitope sequence	Protein (position)	Assay type	Allele	Assay	Ref
1074918	GYQPYRVVVLSF	S (504-515)	T-cell	HLA class I	biological activity activation	[2]
			B-cell		Microarray qualitative binding (negative)	[3]
1334394	YQPYRVVVL	S (505-513)	T-cell	HLA-C*06:02	multimer/tetramer qualitative binding	[4]
51999	QPYRVVVLSF	S (506-515)	T-cell	HLA-B*07:02	biological activity activation	[5]
			MHC	HLA-B*07:02<br>HLA-B*53:01	purified MHC/competitive/radioactivity dissociation constant KD (~IC50)	[6]
1310747	QPYRVVVLSFELLHA	S (506-520)	T-cell	HLA-DQB1*05:03<br>HLA-DRB1*14:01	biological activity activation	[5]
1323467	QPYRVVVL	S (506-513)		HLA class I, II	ICS IFNγ release	[7]
50166	PYRVVVLSF	S (507-515)	T-cell	HLA-A*24:02	ICS IFNγ release	[8]
			MHC	HLA-A*24:02	cellular MHC/competitive/fluorescence qualitative binding	[8]
			MHC	HLA-A*23:01<br>HLA-A*24:02<br>HLA-A*26:01<br>HLA-A*01:01	purified MHC/competitive/radioactivity dissociation constant KD (~IC50)	[6]
1542166	QPYRVVVLSFELLHAPATVC	S (506-525)	T-cell	HLA class II	3H-thymidine proliferation	[9]

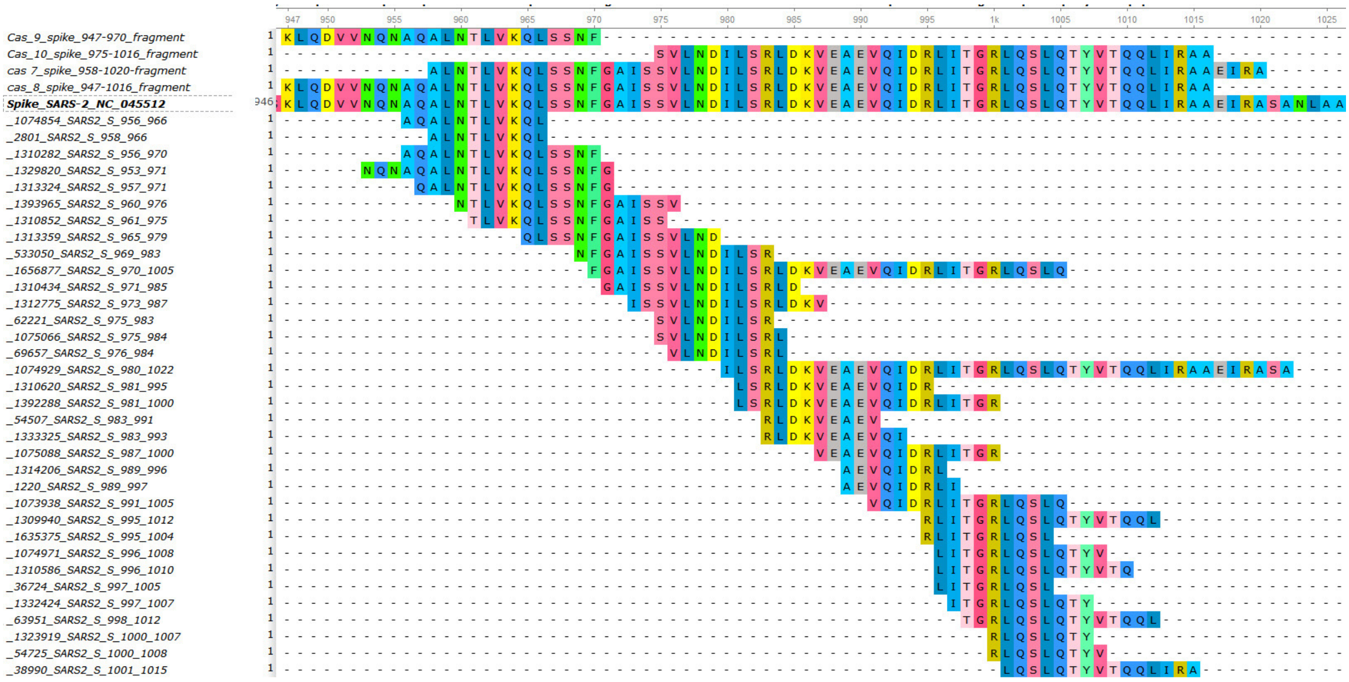

Figure S2. 947-1020 fragment of spike protein aligned with peptides established as immunogenic/MHC molecule binding epitopes in experimental studies. This fragment is a part of cassettes #7, #8, #9, #10. Epitopes' IDs in Immune Epitope Database are indicated on the left. References and additional information regarding these epitopes are listed in Table S4.

This long fragment starts in a heptad repeat 1 and includes a part of central helix [10].

Table S4. Epitopes in 947-1020 fragment of spike protein, deposited in IEDB, additional information and references

Epitope ID	Epitope sequence	Protein (position)	Assay type	Allele	Assay	Ref
1310448	GKLQDVVNQNAQALN	S (946-960)	T-cell	HLA class I, II	ICS IFNγ release	[7]
1329820	NQNAQALNTLVKQLSSNFG	S (953-971)	T-cell	HLA class II	Infectious disease via exposure to SARS-CoV2 (Source Organism) followed by restimulation in vitro	[11]
			MHC	HLA class II	cellular MHC/mass spectrometry ligand presentation	[12]
1074854	AQALNTLVKQL	S (956-966)	T-cell	HLA class I	biological activity activation	[2]
1310282	AQALNTLVKQLSSNF	S (956-970)	T-cell	HLA class II	ELISPOT IFNγ release	[13]
			MHC	HLA class II	cellular MHC/mass spectrometry ligand presentation	[12]
1313324	QALNTLVKQLSSNFG	S (957-971)	T-cell	HLA class II	biological activity activation	[14]
2801	ALNTLVKQL	S (958-966)	T-cell	HLA-A*02:01	multimer/tetramer qualitative binding	[15,16]
			T-cell	HLA-A*02:01	ICS TNFa, IFNγ release	[15,17]
			T-cell	HLA-A*02:01	51 chromium cytotoxicity	[18]
			T-cell	HLA-A*02:01	biological activity activation<br>biological activity degranulation<br>ELISPOT IFNγ release	[15]
			MHC	HLA-A*02:01	cellular MHC/direct/fluorescence qualitative binding	
1393965	NTLVKQLSSNFGAISSV	S (960-976)	T-cell	HLA-DRB3*02:02	ELISPOT IFNγ release	[19]

Epitope ID	Epitope sequence	Protein (position)	Assay type	Allele	Assay	Ref
			MHC	HLA-DRA*01:01/ DRB1*04:01	cellular MHC/competitive/fluorescence qualitative binding	[20]
1310852	TLVKQLSSNFGAISS	S (961-975)	T-cell	HLA class II	ELISPOT IFNγ release	[13]
1313359	QLSSNFGAISSVLND	S (965-979)	T-cell	HLA class II	ELISA IFNγ release	[11]
			B-cell		ELISA qualitative binding	[21]
533050	NFGAISSVLNDILSR	S (969-983)	T-cell	HLA class II	biological activity activation	[14]
1656877	FGAISSVLNDILSRLDKVEAEVQIDRLITGRLQSLQ	S (970-1005)	T-cell	HLA class I	biological activity activation	[22]
1310434	GAISSVLNDILSRLD	S (971-985)	T-cell	HLA class II	ELISPOT IFNγ release	[13]
			B-cell		ELISA qualitative binding	[21]
1312775	ISSVLNDILSRLDKV	S (973-987)	T-cell	HLA class II	biological activity activation	[14]
			B-cell		ELISA qualitative binding	[21]
62221	SVLNDILSR	S (975-983)	T-cell	HLA-A*68:01	biological activity activation	[5]
			MHC	HLA-A*11:01 HLA-A*68:01 HLA-A*31:01	purified MHC/competitive/radioactivity dissociation constant KD (~IC50)	[6]
1075066	SVLNDILSRL	S (975-984)	T-cell	HLA class I	biological activity activation	[2]
69657	VLNDILSRL	S (976-984)	MHC	HLA-A*02:01	cellular MHC/direct/fluorescence qualitative binding	[8,15,18,23–27]
			T-cell	HLA-A*02:01	multimer/tetramer qualitative binding ICS TNFα, IFNγ release ELISPOT IFNγ release biological activity degranulation 51 chromium cytotoxicity	[4,15,16,18,28,29]
1074929	ILSRLDKVEAEVQIDRLITGRLQSLQTYVTQQLIRAAEIRASA	S (980-1022)	T-cell	HLA class I	biological activity activation	[2]
1310620	LSRLDKVEAEVQIDR	S (981-995)	T-cell	HLA class II	3H-thymidine proliferation	[9]
			B-cell		ELISA qualitative binding	[21]
1392288	LSRLDKVEAEVQIDRLITGR	S (981-1000)	T-cell	HLA class II	3H-thymidine proliferation	[9]
54507	RLDKVEAEV	S (983-991)	T-cell	HLA-A*02:01	biological activity activation CFSE proliferation ELISPOT IFNγ release ICS IFNγ release multimer/tetramer qualitative binding	[2,4,8,30,31]
			MHC	HLA-A*02:01 HLA-A*02:02 HLA-A*02:03 HLA-A*02:06 HLA-A*02:07 HLA-A*68:02	cellular MHC/competitive/fluorescence qualitative binding purified MHC/competitive/radioactivity dissociation constant KD (~IC50) purified MHC/competitive/radioactivity half maximal inhibitory concentration (IC50)	[6,8,27,32]
1333325	RLDKVEAEVQI	S (983-993)	T-cell	HLA-A*02:01	multimer/tetramer qualitative binding	[4]
1075088	VEAEVQIDRLITGR	S (987-1000)	T-cell	HLA class I	biological activity activation	[2]
1220	AEVQIDRLI	S (989-997)	T-cell	HLA-B*44:02 HLA-B*44:03	biological activity activation multimer/tetramer qualitative binding	[2,33]

Epitope ID	Epitope sequence	Protein (position)	Assay type	Allele	Assay	Ref
			MHC	HLA-B*44:03<br>HLA-B*44:02<br>HLA-B*40:02<br>HLA-B*45:01	purified MHC/competitive/radioactivity half maximal inhibitory concentration (IC50)	[6]
1314206	AEVQIDRL	S (989-996)	T-cell	HLA-B*40:01	biological activity activation	[5]
1073938	VQIDRLITGRLQSLQ	S (991-1005)	T-cell	HLA class II	ELISPOT IFNγ release	[13]
1309940	RLITGRLQSLQTYVTQQ L	S (995-1012)	T-cell	HLA class I	ICS IFNγ release	[35]
1635375	RLITGRLQSL	S (995-1004)	T-cell	HLA-A*02:01	multimer/tetramer qualitative binding	[31]
1074971	LITGRLQSLQTYV	S (996-1008)	T-cell	HLA class I	biological activity activation	[2]
1310586	LITGRLQSLQTYVTQ	S (996-1010)	T-cell	HLA class II	ELISPOT IFNγ, IL-5 release	[13]
36724	LITGRLQSL	S (997-1005)	T-cell	HLA-A*02:01	multimer/tetramer qualitative binding<br>ICS IFNγ release<br>ELISPOT IFNγ release	[16,26,36–38]
1332424	ITGRLQSLQTY	S (997-1007)	T-cell	HLA-A*01:01	multimer/tetramer qualitative binding	[4]
63951	TGRLQSLQTYVTQQL	S (998-1012)	T-cell	n/d	ELISPOT IFNγ release	[39]
			MHC	HLA-DRB1*01:01	purified MHC/competitive/radioactivity half maximal inhibitory concentration (IC50)	[40]
54725	RLQSLQTYV	S (1000-1008)	T-cell	HLA-A*02:01	surface plasmon resonance (SPR) dissociation constant KD<br>multimer/tetramer qualitative binding<br>ICS TNFα IFNγ release<br>ELISPOT IFNγ release<br>biological activity degranulation<br>biological activity activation	[2,15,16,41,42]
			T-cell	HLA-A*02:03	ICS IFNγ release	[8]
			MHC	HLA-A*02:01<br>HLA-A*02:03<br>HLA-A*02:06<br>HLA-A*68:02	purified MHC/competitive/radioactivity half maximal inhibitory concentration (IC50)<br>purified MHC/competitive/radioactivity dissociation constant KD (~IC50)<br>cellular MHC/competitive/fluorescence qualitative binding<br>x-ray crystallography 3D structure	[6,8,15,25,26,32,36,42]
1323919	RLQSLQTY	S (1000-1007)	T-cell	HLA-B*15:01	biological activity activation	[5]
38990	LQSLQTYVTQQLIRA	S (1001-1015)	T-cell	HLA class II	biological activity activation	[14]
			MHC	HLA-DRB1*01:01	purified MHC/competitive/radioactivity dissociation constant KD (~IC50)	[40]

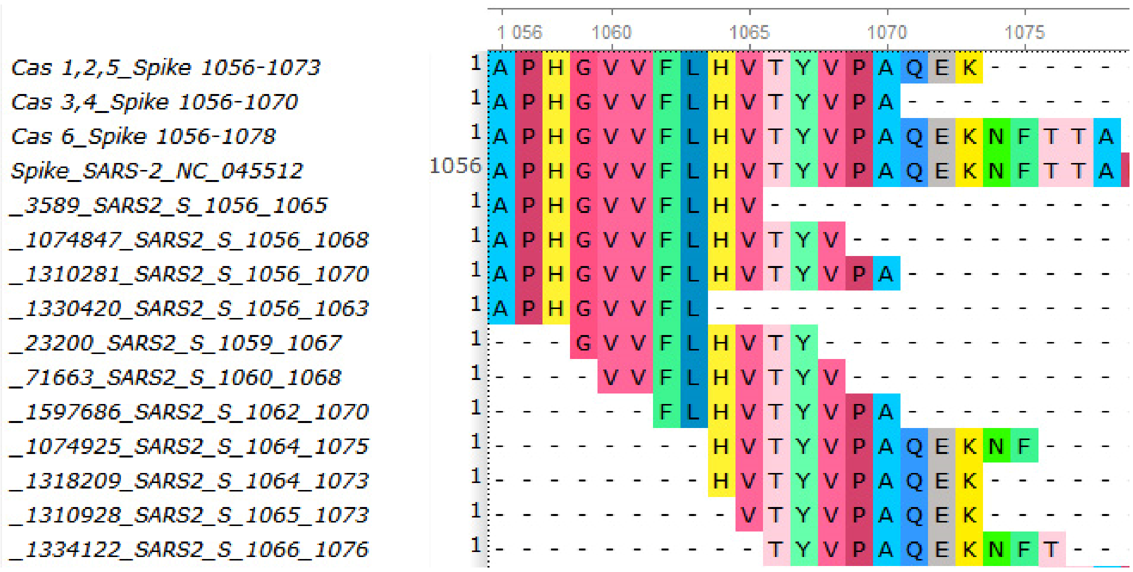

Figure S3. 1056-1078 fragment of spike protein aligned with peptides established as immunogenic/MHC molecule binding epitopes in experimental studies. This fragment is a part of cassettes #1-6. Epitopes' IDs in Immune Epitope Database are indicated on the left. References and additional information regarding these epitopes are listed in Table S5.

This fragment corresponds to connector domain and flanking regions [10].

Table S5. Epitopes in 1056-1078 fragment of spike protein, deposited in IEDB, additional information and references.

Epitope ID	Epitope sequence	Protein (position)	Assay type	Allele	Assay	Ref
3589	APHGVVFLHV	S (1056-1065)	T-cell	HLA-B*07:02	multimer/tetramer qualitative binding	[4]
			MHC	HLA-B*54:01<br>HLA-B*07:02<br>HLA-B*35:01<br>HLA-B*53:01	purified MHC/competitive/radioactivitydissociation constant KD (~IC50)	[6]
1074847	APHGVVFLHVTYV	S 1056-1068)	T-cell	HLA class I	biological activity activation	
1310281	APHGVVFLHVTYVPA	S (1056-1070)	T-cell	HLA class II	biological activity activation ELISPOT IFNγ release	[5,13]
			T-cell	HLA-DRB1*12:01	biological activity activation	[5]
1330420	APHGVVFL	S (1056-1063)	T-cell	HLA-B*07:02	multimer/tetramer qualitative binding	[43]
23200	GVVFLHVTY	S (1059-1067)	T-cell	HLA-A*32:01	biological activity activation	[5]
			MHC	HLA-A*11:01	purified MHC/competitive/radioactivity dissociation constant KD (~IC50)	[6]
71663	VVFLHVTYV	S (1060-1068)	T-cell	HLA-A*02:01	biological activity activation<br>ELISA IFNγ release<br>ICS IFNγ release<br>multimer/tetramer qualitative binding	[4,5,17,26,43]
			MHC	HLA-A*02:01<br>HLA-A*02:03<br>HLA-A*02:06<br>HLA-A*68:02	purified MHC/competitive/radioactivity half maximal inhibitory concentration (IC50)<br>purified MHC/competitive/radioactivity dissociation constant KD (~IC50)<br>cellular MHC/direct/fluorescence qualitative binding	[6,25,26,32,36,44]
1597686	FLHVTYVPA	S (1062-1070)	T-cell	HLA-A*02:03	CFSE proliferation<br>ICS IFNγ release	[8,27]

Epitope ID	Epitope sequence	Protein (position)	Assay type	Allele	Assay	Ref
					multimer/tetramer qualitative binding	
1074925	HVTYVPAQEKNF	S (1064-1075)	T-cell	HLA class I	biological activity activation	[2]
1318209	HVTYVPAQEK	S (1064-1073)	T-cell	HLA-A*68:01	biological activity activation	[5]
1310928	VTYVPAQEK	S (1065-1073)	T-cell	HLA-A*30:01<br>HLA-A*03:01<br>HLA-A*11:01<br>HLA class II	biological activity activation<br>ICS IFNγ release	[5,8]
			MHC	HLA-A*03:01<br>HLA-A*11:01	cellular<br>MHC/competitive/fluorescence<br>qualitative binding<br>purified MHC qualitative binding	[8,45]
1334122	TYVPAQEKNFT	S (1066-1076)	T-cell	HLA-A*24:02	multimer/tetramer qualitative binding	[4]

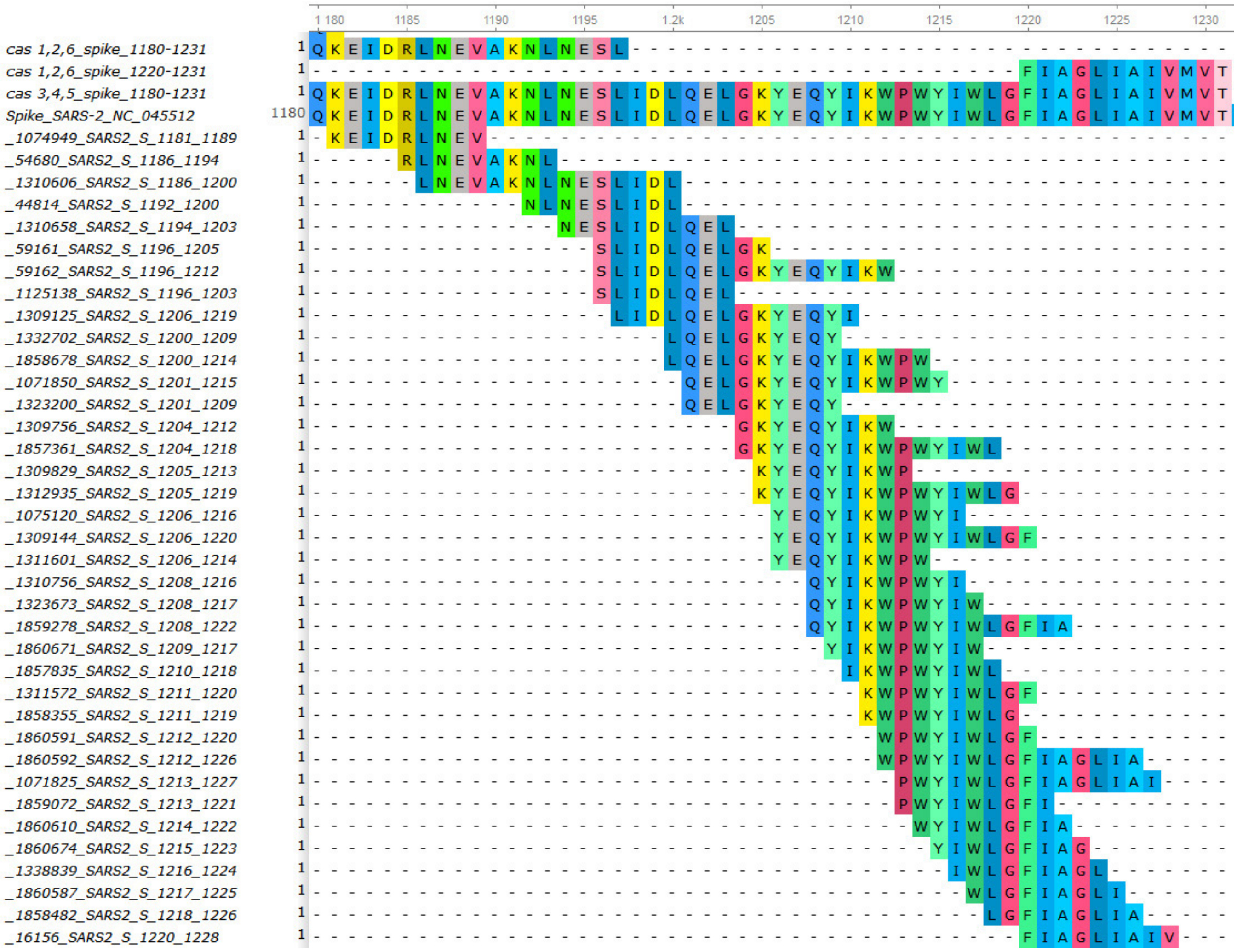

Figure S4. 1180-1231 fragment of spike protein aligned with peptides established as immunogenic/MHC molecule binding epitopes in experimental studies. This fragment is a part of cassettes #1-6. Epitopes' IDs in Immune Epitope Database are indicated on the left. References and additional information regarding these epitopes are listed in Table S6.

Table S6. Epitopes in 1056-1078 fragment of spike protein, deposited in IEDB, additional information and references

Epitope ID	Epitope sequence	Protein (position)	Assay type	Allele	Assay	Ref
1074949	KEIDRLNEV	S (1181-1189)	T-cell	HLA class I<br>HLA-B*44:03<br>HLA-B*44:02<br>HLA-B*40:01	biological activity activation	[2,5]
54680	RLNEVAKNL	S (1186-1194)	T-cell	HLA-A*02:01	multimer/tetramer qualitative binding<br>ICS IFNγ release<br>ELISPOT IFNγ release<br>51 chromium cytotoxicity	[16,25, 28,36,37,46]
			MHC	HLA-A*02:01	cellular MHC/direct/fluorescence qualitative binding<br>purified MHC/direct/fluorescence 50% dissociation temperature	[25,47]
1310606	LNEVAKNLNESLIDL	S (1186-1200)	T-cell	HLA class II	ELISPOT<br>IFNγ release	[13]
44814	NLNESLIDL	S (1192-1200)	T-cell	HLA-A*02:01	51 chromium cytotoxicity<br>ELISPOT IFNγ release<br>multimer/tetramer qualitative binding<br>High throughput multiplexed assay T cell binding	[2,16,18,28]
			MHC	HLA-A*02:01	cellular MHC/direct/fluorescence qualitative binding<br>cellular MHC/mass spectrometry ligand presentation	[18,23, 25,26,48]
1310658	NESLIDLQEL	S (1194-1203)	T-cell	HLA-B*40:01	biological activity activation	[5]
59161	SLIDLQELGK	S (1196-1205)	T-cell	HLA-A*11:01<br>HLA class II	ICS IFNγ release	[8]
			MHC	HLA-A*11:01<br>HLA-A*68:01<br>HLA-A*03:01<br>HLA-A*31:01<br>HLA-A*33:01	cellular MHC/competitive/fluorescence qualitative binding<br>purified MHC/competitive/radioactivity dissociation constant KD (~IC50)	[6,8]
59162	SLIDLQELGKYEQYIKW	S (1196-1212)	T-cell	HLA class I	High throughput multiplexed assay T cell binding	[2]
1125138	SLIDLQEL	S (1196-1203)	T-cell	HLA-A*02:01	multimer/tetramer qualitative binding	[4]
1332702	LQELGKYEQY	S (1200-1209)	T-cell	HLA-A*01:01	multimer/tetramer qualitative binding	[4]
1858678	LQELGKYEQYIKWPW	S (1200-1214)	T-cell	HLA-A*24:02	ICS IFNγ release<br>ICS TNFα release	[49]
1071850	QELGKYEQYIKWPWY	S (1201-1215)	T-cell	HLA class II	biological activity activation	[5]
			B-cell		ELISA qualitative binding	[21]
1323200	QELGKYEQY	S (1201-1209)	T-cell	HLA-B*44:03<br>HLA-B*44:02	biological activity activation	[5]
1309756	GKYEQYIKW	S (1204-1212)	T-cell	HLA-A*24:02	ICS IFNγ release	[49]
1857361	GKYEQYIKWPWYIWL	S (1204-1218)	T-cell	HLA-A*24:02	ICS IFNγ release	[49]
1309829	KYEQYIKWP	S (1205-1213)	T-cell	HLA-A*24:02	ICS IFNγ release	[49]
			MHC	HLA class II	cellular MHC/mass spectrometry ligand presentation	[12]

Epitope ID	Epitope sequence	Protein (position)	Assay type	Allele	Assay	Ref
1312935	KYEQYIKWPWYIWLG	S (1205-1219)	T-cell	HLA-A*24:02<br>HLA class I	multimer/tetramer qualitative binding<br>High throughput multiplexed assay T cell binding	[2,4]
1075120	YEQYIKWPWYI	S (1206-1216)	T-cell	HLA-A*24:02<br>HLA class I	multimer/tetramer qualitative binding<br>High throughput multiplexed assay T cell binding	[2,4]
1309125	LIDLQELGKYEQYI	S (1206-1219)	T-cell	nd	ELISPOT IFNγ release	[50]
1309144	YEQYIKWPWYIWLGF	S (1206-1220)	T-cell	HLA class II	ELISPOT IFNγ release	[13,50]
1311601	YEQYIKWPW	S (1206-1214)	T-cell	HLA-A*24:02<br>HLA-B*44:02<br>HLA-B*44:03	biological activity activation<br>ICS IFNγ release<br>ICS TNFα release	[5,49,51]
1310756	QYIKWPWYI	S (1208-1216)	T-cell	HLA-A*24:02	biological activity activation<br>ELISA IFNγ release<br>ELISPOT IFNγ release<br>ICS IFNγ release<br>ICS TNFα release<br>multimer/tetramer qualitative binding	[4,5,17,33,43,46,49,52–57]
1323673	QYIKWPWYIW	S (1208-1217)	T-cell	HLA-A*23:01	biological activity activation	[5]
1859278	QYIKWPWYIWLGFIA	S (1208-1222)	T-cell	HLA-A*24:02	ICS IFNγ, TNFα release	[49]
1860671	YIKWPWYIW	S (1209-1217)	T-cell	HLA-A*24:02	ICS IFNγ, TNFα release	[49]
1857835	IKWPWYIWL	S (1210-1218)	T-cell	HLA-A*24:02	ICS IFNγ, TNFα release	[49]
1311572	KWPWYIWLGF	S (1211-1220)	T-cell	HLA-A*24:02	ICS IFNγ, TNFα release	[17,51]
1858355	KWPWYIWLG	S (1211-1219)	T-cell	HLA-A*24:02	ICS IFNγ, TNFα release	[49]
1860591	WPWYIWLGF	S (1212-1220)	T-cell	HLA-A*24:02	ICS IFNγ, TNFα release	[49]
1860592	WPWYIWLGFIAGLIA	S (1212-1226)	T-cell	HLA-A*24:02	ICS IFNγ, TNFα release	[49]
1071825	PWYIWLGFIAGLIAI	S (1213-1227)	T-cell	nd	ELISPOT IFNg release	[58]
			B-cell		ELISA qualitative binding	[21]
1859072	PWYIWLGFI	S (1213-1221)	T-cell	HLA-A*24:02	ICS IFNγ, TNFα release	[49]
1860610	WYIWLGFIA	S (1214-1222)	T-cell	HLA-A*24:02	ICS IFNγ, TNFα release	[49]
1860674	YIWLGFIAG	S (1215-1223)	T-cell	HLA-A*24:02	ICS IFNγ, TNFα release	[49]
1338839	IWLGFIAGL	S (1216-1224)	T-cell	HLA-A*24:02	ICS IFNγ, TNFα release	[49]
1860587	WLGFIAGLI	S (1217-1225)	T-cell	HLA-A*24:02	ICS IFNγ, TNFα release	[49]
1858482	LGFIAGLIA	S (1218-1226)	T-cell	HLA-A*24:02	ICS IFNγ, TNFα release	[49]
16156	FIAGLIAIV	S (1220-1228)	T-cell	HLA-A*02:01	biological activity activation<br>in vivo assay cytotoxicity<br>biological activity degranulation<br>ELISA IFNγ release<br>ELISPOT IFNγ release<br>High throughput multiplexed assay T cell binding<br>ICS IFNγ, TNFα release<br>multimer/tetramer qualitative binding	[2,4,8,15,16,28,38,41,59–62]
			MHC	HLA-A*02:01<br>HLA-A*02:02<br>HLA-A*02:03<br>HLA-A*02:06<br>HLA-A*68:02	purified MHC/competitive/radioactivity dissociation constant KD (~IC50)<br>cellular MHC/direct/fluorescence qualitative binding	[6,8,15,32,38]

Epitope ID	Epitope sequence	Protein (position)	Assay type	Allele	Assay	Ref
					purified MHC/competitive/radioactivity half maximal inhibitory concentration (IC50)	

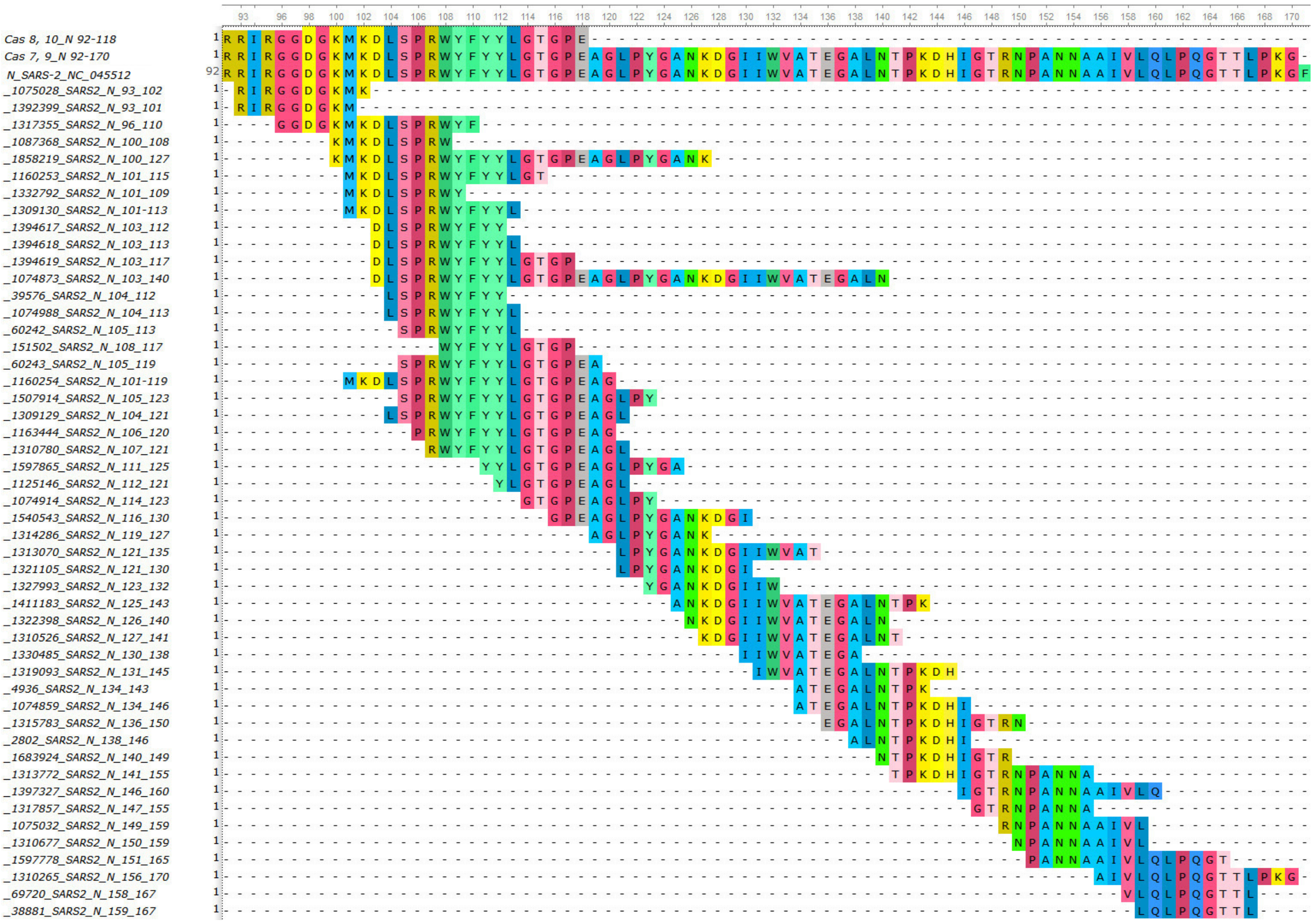

Figure S5. 92-170 fragments of nucleocapsid protein aligned with peptides established as immunogenic/MHC molecule binding epitopes in experimental studies. This fragment is a part of cassette #7-10. Epitopes' IDs in Immune Epitope Database are indicated on the left. References and additional information regarding these epitopes are listed in Table S7. This fragment is a part of a fragment of RNA-binding domain [64].

Table S7. Epitopes in 92-170 fragment of nucleocapsid protein, deposited in IEDB, additional information and references

Epitope ID	Epitope sequence	Protein (position)	Assay type	Allele	Assay	Ref
1325864	TRRIRGGDGKMKDLS	N (91-105)	T-cell	HLA class II	biological activity activation ICS IFNγ release	[5,65]
1075028	RIRGGDGKMK	N (93-102)	T-cell	HLA class I	High throughput multiplexed assay T cell binding	[2]
1392399	RIRGGDGKM	N (93-101)	T-cell	HLA-B*07:02	biological activity activation ICS IFNγ release	[66–68]
1317355	GGDGKMKDLSPRWYF	N (96-110)	T-cell	HLA class II	biological activity activation ICS IFNγ release	[5,65]
			B-cell		ELISA qualitative binding	[21]
1087368	KMKDLSPRW	N (100-108)	T-cell	HLA-B*57:01 HLA class I	biological activity activation	[2,5]

					High throughput multiplexed assay T cell binding	
1858219	KMKDLSPRWYFYYLGTGPEAGLPYGANK	N (100-127)	T-cell	HLA class I, II	biological activity activation	[69]
1160253	MKDLSPRWYFYYLGT	N (101-115)	T-cell	HLA class II	biological activity activation<br>ELISPOT IFNγ release<br>ICS IFNγ release	[5,65,70]
1332792	MKDLSPRWY	N (101-109)	T-cell	HLA-C*07:01	multimer/tetramer qualitative binding	[4]
1074873	DLSPRWYFYYLGTGPEAGLPYGANKDGIIWVATEGALN	N (103-140)	T-cell	HLA class I	High throughput multiplexed assay T cell binding	[2]
1394617	DLSPRWYFYY	N (103-112)	T-cell	HLA-A2<br>HLA-A29	ELISPOT IFNγ release	[71]
1394618	DLSPRWYFYYL	N (103-113)	T-cell	HLA-A2<br>HLA-A29	ELISPOT IFNγ release	[71]
1394619	DLSPRWYFYYLGTGP	N (103-117)	T-cell	HLA-A2<br>HLA-A29<br>HLA class I, II	ELISPOT IFNγ release<br>biological activity degranulation<br>ICS IFNγ release	[71]
1074988	LSPRWYFYYL	N (104-113)	T-cell	HLA-A*24:02<br>HLA class I	ICS IFNγ release<br>High throughput multiplexed assay T cell binding	[2,8]
1309129	LSPRWYFYYLGTGPEAGL	N (104-121)	T-cell	HLA-B*07:02<br>HLA-C*07:02<br>HLA-A*02:01	ELISPOT IFNγ release	[50]
60242	SPRWYFYYL	N (105-113)	T-cell	HLA-B*07:02<br>HLA-B*08:01<br>HLA-B35<br>HLA-A*02:01	biological activity activation<br>biological activity degranulation<br>ELISA IFNγ release<br>ELISPOT IFNγ release<br>ICS CCL4/MIP-1b release<br>ICS IFNγ, IL-2, TNFα release<br>in vitro assay cytotoxicity<br>multimer/tetramer qualitative binding	[4,5,17,43,48,50,50,51,53,56,66–68,71,72]
			MHC	HLA-B*07:02<br>HLA-B*54:01<br>HLA-B*51:01<br>HLA-B*53:01	purified MHC/competitive/radioactivity dissociation constant KD (~IC50)<br>purified MHC/direct/fluorescence 50% dissociation temperature	[6,66]
60243	SPRWYFYYLGTGPEA	N (105-119)	T-cell	HLA class II	ELISA IFNγ release	[11]
1507914	SPRWYFYYLGTGPEAGLPY	N (105-123)	T-cell	HLA class II	ELISA IFNγ release	[11]
1163444	PRWYFYYLGTGPEAG	N (106-120)	T-cell	HLA class II<br>HLA-DQB1*05:03<br>HLA-DQB1*02:01<br>HLA-DQA1*05:01/DQB1*02:01<br>HLA-DQA1*05:01/DQB1*02:01<br>HLA-DQA1*05:01/DQB1*02:01<br>HLA-DRB1*01:01<br>HLA-DRB1*08:02	ICS IFNγ release<br>biological activity activation<br>ELISPOT IFNγ release	[5,65,70]

				HLA-DRB1*11:01<br>HLA-DRB3*01:01<br>HLA-DRB3*02:02<br>HLA-DRB1*04:01<br>HLA-DRB1*04:05<br>HLA-DRB1*12:01<br>HLA-DQA1*03:01/DQB1*03:02<br>HLA-DRB1*07:01<br>HLA-DRB1*15:01<br>HLA-DRB5*01:01<br>HLA-DQB1*05:01<br>HLA-DPB1*02:01<br>HLA-DQA1*01:01/DQB1*05:01<br>HLA-DRB1*09:01		
1310780	RWYFYYLGTGPEAGL	N (107-121)	T-cell	HLA-DR	biological activity activation<br>ELISPOT IFNγ release<br>ICS IFNγ, IL-2, TNFα release	[52,57,73]
151502	WYFYYLGTGP	N (108-117)	T-cell	nd	ELISPOT IFNγ release	[71]
1597865	YYLGTGPEAGLPYGA	N (111-125)	T-cell	HLA class II	ICS IFNγ release	[65]
1125146	YLGTGPEAGL	N (112-121)	T-cell	HLA-A*02:01	biological activity activation<br>ICS IFNγ release<br>multimer/tetramer qualitative binding	[23,41]
1074914	GTGPEAGLPY	N (114-135)	T-cell	HLA class I	High throughput multiplexed assay T cell binding	[2]
1540543	GPEAGLPYGANKDGI	N (116-130)	T-cell	HLA class II	ICS IFNγ release	[65]
1314286	AGLPYGANK	N (119-127)	T-cell	HLA-A*30:01	biological activity activation	[5]
1313070	LPYGANKDGIIWVAT	N (121-135)	T-cell	HLA class II<br>HLA-DQA1*05:01/DQB1*02:01<br>HLA-DQA1*05:01/DQB1*03:01<br>HLA-DRB1*01:01<br>HLA-DRB4*01:01<br>HLA-DRB1*08:02<br>HLA-DRB1*11:01<br>HLA-DRB3*01:01<br>HLA-DRB3*02:02<br>HLA-DRB1*04:01<br>HLA-DRB1*04:05<br>HLA-DRB1*12:01<br>HLA-DRB1*09:01<br>HLA-DQA1*01:02/DQB1*06:02<br>HLA-DQA1*03:01/DQB1*03:02<br>HLA-DRB1*07:01<br>HLA-DRB1*15:01<br>HLA-DRB5*01:01	ICS IFNγ release<br>biological activity activation<br>ELISPOT IFNγ release	[5,65,70]

				HLA-DPA1*01:03/DPB1*04:01		
1321105	LPYGANKDGI	N (121-130)	T-cell	HLA-B*51:01	biological activity activation	[5]
1327993	YGANKDGIIW	N (123-132)	T-cell	HLA-B*57:01	biological activity activation	[5]
1411183	ANKDGIIWVATEGALNTPK	N (125-140)	T-cell	HLA class II	ELISA IFNγ release	[11]
1322398	NKDGIIWVATEGALN	N (126-140)	T-cell	HLA-DRB1*01:01<br>HLA-DRB1*03:01<br>HLA-DRB1*04:01<br>HLA-DRB1*04:04<br>HLA-DRB1*04:05<br>HLA-DRB1*07:01<br>HLA-DRB1*08:02<br>HLA-DRB1*09:01<br>HLA-DRB1*11:01<br>HLA-DRB1*15:01<br>HLA-DRB3*01:01<br>HLA-DRB3*02:02<br>HLA-DRB4*01:01<br>HLA-DRB5*01:01<br>HLA-DQB1*02:01<br>HLA-DQB1*02:02<br>HLA-DQB1*03:02<br>HLA-DQB1*05:03<br>HLA-DPB1*02:01<br>HLA-DQA1*05:01/DQB1*02:01<br>HLA-DQA1*05:01/DQB1*03:01<br>HLA-DQA1*03:01/DQB1*03:02<br>HLA-DQA1*01:02/DQB1*06:02<br>HLA-DPA1*01:03/DPB1*04:01	ICS IFNγ release<br>biological activity activation<br>ELISPOT IFNγ release	[5,65]
			B-cell		ELISAqualitative binding	[21]
1310526	KDGIIWVATEGALNT	N (127-141)	T-cell	HLA-DR	biological activity activation<br>ELISPOT IFNγ release<br>ICS IFNγ, TNFα release	[52,57]
1330485	IIWVATEGA	N (130-138)	T-cell	HLA-A*02:01	biological activity degranulation<br>ICS IFNγ, IL-2, TNFα release	[74]
1319093	IWVATEGALNTPKDH	N (131-145)	T-cell	HLA class II	biological activity activation<br>ICS IFNγ release	[5,65]
4936	ATEGALNTPK	N (134-143)	T-cell	HLA-A*11:01	biological activity activation<br>ELISA IFNγ release<br>ELISPOT IFNγ release<br>ICS IFNγ release<br>in vitro assay cytotoxicity	[5,17,53,57,68]
			MHC	HLA-A*11:01<br>HLA-A*68:01<br>HLA-A*03:01<br>HLA-A*31:01	cellular MHC/mass spectrometry ligand presentation<br>purified MHC/competitive/radioactivity dissociation constant KD (~IC50)	[6,75]

1074859	ATEGALNTPKDHI	N (134-146)	T-cell	HLA class I	High throughput multiplexed assay T cell binding	[2]
1315783	EGALNTPKDHIGTRN	N (136-150)	T-cell	HLA class II	biological activity activation<br>ICS IFNγ release	[5,65]
			B-cell		ELISA qualitative binding	[21]
2802	ALNTPKDHI	N (138-146)	T-cell	HLA-A*02:01	ELISPOT IFNγ release<br>ICS IFNγ release	[76–78]
			MHC	HLA-A*02:01	cellular MHC/direct/fluorescence qualitative binding<br>purified MHC/direct/fluorescence 50% dissociation temperature	[26,77,78]
1683924	NTPKDHIGTR	N (140-149)	T-cell	HLA-A*33:03	ICS IFNγ release	[8]
1313772	TPKDHIGTRNPANNA	N (141-155)	T-cell	HLA class II	ICS IFNγ release	[65]
1397327	IGTRNPANNAAIVLQ	N (143-160)	T-cell	nd	ELISPOT IFNγ release	[65]
			B-cell		Microarray qualitative binding	[79]
1317857	GTRNPANNA	N (147-155)	T-cell	HLA-A*30:01	biological activity activation	[5]
1075032	RNPANNAAIVL	N (149-159)	T-cell	HLA class I	High throughput multiplexed assay T cell binding	[2]
1310677	NPANNAAIVL	N (150-159)	T-cell	HLA-B*07:02	biological activity activation<br>ICS IFNγ release	[5,65]
1597778	PANNAAIVLQLPQGT	N (151-165)	T-cell	HLA class II	ICS IFNγ release	[65]
1310265	AIVLQLPQGTTLPKG	N (156-170)	T-cell	HLA-DR	ELISPOT IFNγ release	[52,57,65]
			B-cell		biological activity agglutination<br>ELISA qualitative binding<br>Microarray qualitative binding	[21,79,80]
69720	VLQLPQGTTL	N (158-167)	T-cell	HLA-A*02:01	multimer/tetramer qualitative binding	[4]
1075095	VLQLPQGTTLPKGFYA	N (158-173)	T-cell	HLA class I	High throughput multiplexed assay T cell binding	[2]
			B-cell		Microarray qualitative binding	[81]
38881	LQLPQGTTL	N (159-167)	T-cell	HLA-A*02:01	ELISPOT IFNγ release<br>ICS IFNγ, TNFα release	[76–78]
			MHC	HLA-A*02:01	cellular MHC/direct/fluorescence qualitative binding<br>purified MHC/direct/fluorescence 50% dissociation temperature	[76,78]

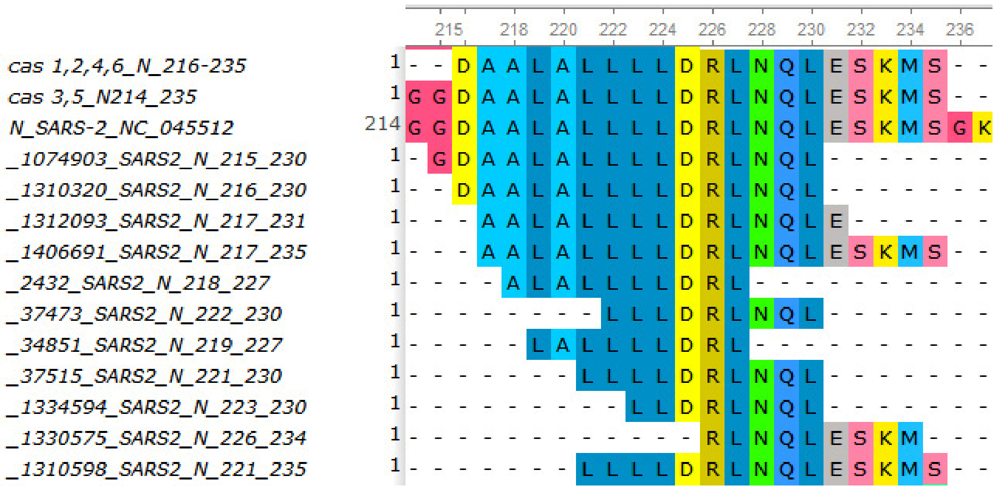

Figure S6. 214-235 fragment of nucleocapsid protein aligned with peptides established as immunogenic/MHC molecule binding epitopes in experimental studies. This fragment is a part of cassettes #1-6. Epitopes' IDs in Immune Epitope Database are indicated on the left. References and additional information regarding these epitopes are listed in Table S8. This fragment is part of the linker connecting two domains, right after the SR-rich region [82].

Table S8. Epitopes in 214-235 fragment of nucleocapsid protein, deposited in IEDB, additional information and references

Epitope ID	Epitope sequence	Protein (position)	Assay type	Allele	Assay	Ref
1074903	GDAALALLLLDRLNQL	N (215-230)	T-cell	HLA class I	High throughput multiplexed assay<br>T cell binding	[2]
			B-cell		Microarray qualitative binding	[81]
1310320	DAALALLLLDRLNQL	N (216-230)	T-cell	HLA-DQB1*05:03<br>HLA class II<br>HLA-DRB1*15:01<br>HLA-DRB1*12:01<br>HLA-DQB1*06:02<br>HLA-DRB1*03:01<br>HLA-DRB1*14:01<br>HLA-DPA1*01:03/DPB1*04:01<br>HLA-DQA1*05:01/DQB1*03:01<br>HLA-DQA1*03:01/DQB1*03:02<br>HLA-DQA1*01:01/DQB1*05:01<br>HLA-DQA1*01:02/DQB1*06:02<br>HLA-DRB1*04:05<br>HLA-DRB1*08:02<br>HLA-DRB1*09:01<br>HLA-DRB1*13:02<br>HLA-DQB1*05:03<br>HLA-DRB1*11:01<br>HLA-DRB5*01:01<br>HLA-DRB1*03:01<br>HLA-DRB1*04:01<br>HLA-DRB3*01:01<br>HLA-DRB4*01:01<br>HLA-DRB1*14:01	biological activity activation<br>ELISPOT IFNγ release<br>ELISPOT IL-5 release<br>ICS IFNγ release	[5,13, 65]

Epitope ID	Epitope sequence	Protein (position)	Assay type	Allele	Assay	Ref
				HLA-DRB1*01:01<br>HLA-DQA1*05:01/DQB1*02:01<br>HLA-DPB1*02:01		
			MHC	HLA-DRB1*11:01<br>HLA-DRB3*01:01<br>HLA-DRB1*13:02<br>HLA-DRB3*01:01<br>HLA-DRB4*01:01<br>HLA-DRB1*15:01<br>HLA-DRB1*04:05<br>HLA-DRB4*01:01<br>HLA-DRB1*12:01<br>HLA-DQA1*03:01/DQB1*03:02<br>HLA-DRB1*15:01<br>HLA-DRB1*08:02<br>HLA-DRB1*04:05<br>HLA-DRB1*08:02<br>HLA-DPB1*02:01<br>HLA-DRB1*01:01<br>HLA-DQA1*01:02/DQB1*06:02<br>HLA-DQA1*05:01/DQB1*02:01<br>HLA-DRB1*03:01<br>HLA-DRB1*12:01<br>HLA-DRB1*13:02<br>HLA-DPA1*01:03/DPB1*04:01<br>HLA-DRB1*04:01<br>HLA-DQA1*01:01/DQB1*05:01<br>HLA-DRB1*09:01<br>HLA-DRB5*01:01<br>HLA-DRB1*09:01<br>HLA-DRB5*01:01<br>HLA-DQA1*05:01/DQB1*03:01<br>HLA-DRB1*03:01<br>HLA-DRB1*07:01	purified MHC/competitive/radioactivity<br>half maximal inhibitory concentration (IC50)	[5,65]
1312093	AALALLLLDRLNQLE	N (217-231)	T-cell	HLA-DRB1*11:01<br>HLA class II	biological activity activation<br>cytometric bead array IL-10, IL-13, IL-4, IL-5, TNFα release<br>ELISA IFNγ release	[11,67]
			B-cell		Microarray qualitative binding	[34,84]
1406691	AALALLLLDRLNQLESKMS	N (217-235)	T-cell	HLA class II	ELISA IFNγ release	[11]
2432	ALALLLLDRL	N (218-227)	T-cell	HLA-A2	ELISPOT IFNγ release	[85]
34851	LALLLLDRL	N (219-227)	T-cell	HLA-A*02:01<br>HLA-A*02:06<br>HLA class II	ELISPOT IFNγ release<br>ICS IFNγ, TNFα release<br>in vitro assay cytotoxicity	[8,30,51,56,62,76–78,86]

Epitope ID	Epitope sequence	Protein (position)	Assay type	Allele	Assay	Ref
			MHC	HLA-A*02:01	cellular MHC/direct/fluorescence qualitative binding<br>purified MHC/direct/fluorescence 50% dissociation temperature	[76–78]
37515	LLLLDRLNQL	N (221-230)	T-cell	HLA-A*02:01	ELISPOT IFNγ release<br>ICS IFNγ, TNFα release<br>multimer/tetramer qualitative binding	[4,57,87,88]
			MHC	HLA-A*02:01	cellular MHC/direct/fluorescence qualitative binding<br>purified MHC/direct/fluorescence qualitative binding	[36,83]
1310598	LLLLDRLNQLESKMS	N (221-235)	T-cell	HLA class II<br>HLA-DR<br>HLA-DRB1*14:01<br>HLA-DRB1*15:01<br>HLA-DRB1*03:01<br>HLA-DRB1*12:01<br>HLA-DQA1*03:01/DQB1*03:02<br>HLA-DQA1*01:01/DQB1*05:01<br>HLA-DQA1*01:02/DQB1*06:02<br>HLA-DRB1*07:01<br>HLA-DRB1*08:02<br>HLA-DRB1*11:01<br>HLA-DRB1*13:02<br>HLA-DRB4*01:01<br>HLA-DRB1*11:01<br>HLA-DRB1*01:01<br>HLA-DPA1*01:03/DPB1*04:01<br>HLA-DRB1*09:01<br>HLA-DRB3*01:01<br>HLA-DQA1*05:01/DQB1*02:01<br>HLA-DRB1*04:05<br>HLA-DRB1*04:01<br>HLA-DRB5*01:01<br>HLA-DPB1*02:01<br>HLA-DRB1*12:01	biological activity activation<br>ELISPOT IFNγ release<br>ICS IFNγ, TNFα release	[5,13,52,57,65,73,88]
1858532	LLLLDRLNQLESKMF	N (221-235)	T-cell	HLA-DR	ELISPOT IFNγ release	[88]
37473	LLLDRLNQL	N (222-230)	T-cell	HLA-A*02:01	51 chromium cytotoxicity<br>biological activity activation<br>CFSE proliferation<br>ELISA IFNγ release<br>ICS IFNγ, TNFα release<br>in vitro assay cytotoxicity<br>in vivo assay pathogen burden after challenge	[5,8,23,26,30,41,43,51,53,53,56,62,72,76–

Epitope ID	Epitope sequence	Protein (position)	Assay type	Allele	Assay	Ref
					multimer/tetramer qualitative binding	78,83,88]
			MHC	HLA-A*02:01 HLA-A*02:03	cellular MHC/competitive/fluorescence qualitative binding cellular MHC/direct/fluorescence qualitative binding purified MHC/direct/fluorescence 50% dissociation temperature	[8,8,23,26,36,76–78,83]
1334594	LLDRLNQL	N (223-230)	T-cell	HLA-DR	ICS IL-2 release	[73]
1330575	RLNQLESKM	N (226-234)	T-cell	HLA-A*02:01	ICS IFNγ, TNFα release	[78]

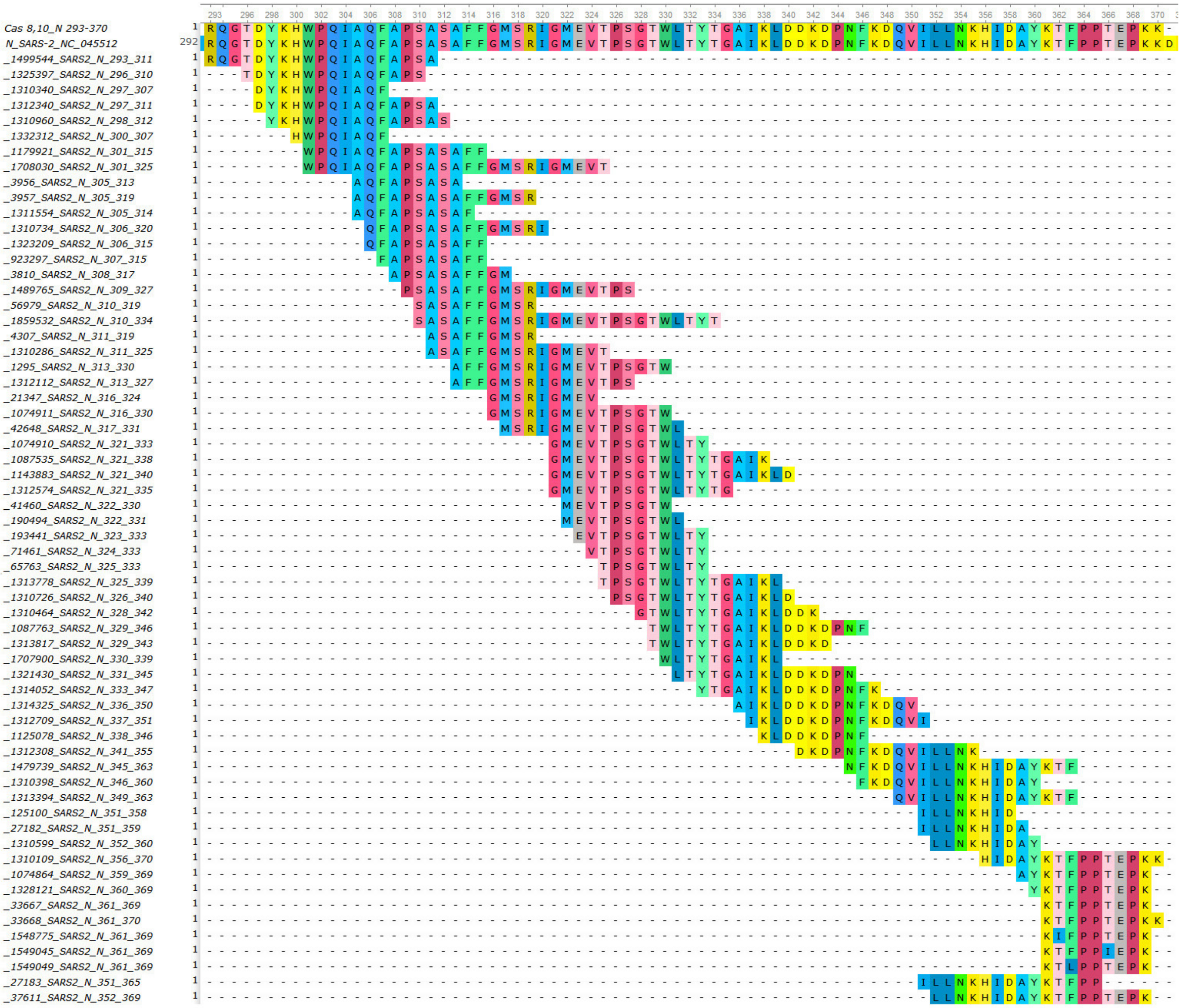

Figure S7. 293-370 fragments of nucleocapsid protein aligned with peptides established as immunogenic/MHC molecule binding epitopes in experimental studies. This fragment is a part of cassettes #8 and #10. Epitopes' IDs in Immune Epitope Database are indicated on the left. References and additional information regarding these epitopes are listed in Table S9. This fragment corresponds to a large fragment of dimerization domain of N [82].

Table S9. Epitopes in 214-235 fragment of nucleocapsid protein, deposited in IEDB, additional information and references

Epitope ID	Epitope sequence	Protein (position)	Assay type	Allele	Assay	Ref
1156855	LIRQGTDYKHWPQIA	N (291-305)	T-cell	HLA class II	biological activity activation<br>ICS IFNγ, TNFα release	[5,65,70]
1499544	RQGTDYKHWPQIAQFAPSA	N (293-311)	T-cell	HLA class II	ELISA IFNγ release	[11]
1325397	TDYKHWPQIAQFAPS	N (296-310)	T-cell	HLA class II	biological activity activation<br>ICS IFNγ release	[5,65]
			B-cell		ELISA qualitative binding	[21]
1310340	DYKHWPQIAQF	N (297-307)	T-cell	HLA-A*24:02	ELISPOT IFNγ release<br>ICS IFNγ, TNFα release	[52,57]
1312340	DYKHWPQIAQFAPSA	N (297-311)	T-cell	HLA class II	ELISA IFNγ release	[11]
1310960	YKHWPQIAQFAPSAS	N (298-312)	T-cell	HLA-DR	ELISPOT IFNγ release<br>ICS IFNγ, TNFα release	[52,57]
1332312	HWPQIAQF	N (300-307)	T-cell	HLA-A*24:02	multimer/tetramer qualitative binding	[4]
1708030	WPQIAQFAPSASAFFGMSRIGMEVT	N (301-325)	T-cell	HLA class I	biological activity activation	[22]
3956	AQFAPSASA	N (305-313)	T-cell	HLA-A*02:01	ICS IFNγ release	[17,29]
			MHC	HLA-A*02:01<br>HLA-A*02:02<br>HLA-A*02:03<br>HLA-A*02:06<br>HLA-B*52:01	cellular MHC/direct/fluorescence qualitative binding<br>purified MHC/competitive/radioactivity dissociation constant KD (~IC50)<br>purified MHC/competitive/radioactivity half maximal inhibitory concentration (IC50)	[6,24,32]
3957	AQFAPSASAFFGMSR	N (305-319)	T-cell	HLA class I, II	ELISA IFNγ release<br>ICS IFNγ release<br>High throughput multiplexed assay T cell binding	[2,89,90]
1311554	AQFAPSASAF	N (305-314)	T-cell	HLA-B*15:01<br>HLA-A*24:02	ICS IFNγ release<br>multimer/tetramer qualitative binding	[4,51,68]
1310734	QFAPSASAFFGMSRI	N (306-320)	T-cell	HLA-DRB1*07:01<br>HLA class II	biological activity activation<br>ICS IFNγ release	[5,65]
			B-cell		ELISA qualitative binding	[21]
1323209	QFAPSASAFF	N (306-315)	T-cell	HLA-A*24:02	biological activity activation	[5]
923297	FAPSASAFF	N (307-315)	T-cell	HLA-B*35:01<br>HLA class I	biological activity activation<br>ELISPOT IFNγ release	[5,62,91]
			MHC	HLA-C*01:02	purified MHC qualitative binding	[45]
3810	APSASAFFGM	N (308-317)	T-cell	HLA-B*07:02	biological activity activation<br>multimer/tetramer qualitative binding	[4,5]
			MHC	HLA-B*07:02<br>HLA-B*35:01<br>HLA-B*53:01<br>HLA-B*51:01<br>HLA-B*81:01	cellular MHC/direct/fluorescence qualitative binding<br>purified MHC/competitive/radioactivity dissociation constant KD (~IC50)	[6,24]
1489765	PSASAFFGMSRIGMEVTPS	N (309-327)	T-cell	HLA class II	ELISA IFNγ release	[11]
56979	SASAFFGMSR	N (310-319)	T-cell	HLA-A*68:01	biological activity activation	[5]

Epitope ID	Epitope sequence	Protein (position)	Assay type	Allele	Assay	Ref
			MHC	HLA-A*11:01 HLA-A*31:01 HLA-A*68:01 HLA-A*33:01 HLA-A*03:01	purified MHC/competitive/radioactivity dissociation constant KD (~IC50) -	[6]
1859532	SASAFFGMSRIGMEVTPSGTWLTYT	N (310-319)	T-cell	HLA class I, HLA class II	biological activity activation ELISPOT IFNγ release	[69]
4307	ASAFFGMSR	N (311-319)	T-cell	HLA-A*11:01 HLA-A*68:01	in vitro assay cytotoxicity biological activity activation	[5,53]
			MHC	HLA-A*11:01 HLA-A*31:01 HLA-A*68:01 HLA-A*03:01	purified MHC/competitive/radioactivity dissociation constant KD (~IC50)	[6]
1310286	ASAFFGMSRIGMEVT	N (311-325)	T-cell	HLA-DRB1*14:01 HLA-DQB1*05:03 HLA-DQB1*05:03 HLA-DR11 HLA-DR	biological activity activation ELISPOT IFNγ release ICS IFNγ release ICS IL-2 release ICS TNF release	[5,52,57,73]
1295	AFFGMSRIGMEVTPSGTW	N (313-330)	T-cell	n/d	ELISPOT IFNγ release	[50,92]
1312112	AFFGMSRIGMEVTPS	N (313-327)	T-cell	HLA class II	ELISPOT IFNγ release	[93]
21347	GMSRIGMEV	N (316-324)	T-cell	HLA-A*02:01	51 chromium cytotoxicity biological activity activation ELISA IFNγ release ICS IFNγ, TNFα release in vitro assay cytotoxicity multimer/tetramer qualitative binding	[4,5,8,26,28,76–78,83,94]
1074911	GMSRIGMEVTPSGTW	N (316-330)	T-cell	HLA class I, HLA class II	High throughput multiplexed assay T cell binding ICS IFNγ release	[2,65]
42648	MSRIGMEVTPSGTWL	N (317-331)	T-cell	HLA class I	ELISPOT IFNγ release ELISPOT TNFα release	[93]
			MHC	HLA-DRB1*01:01	purified MHC/competitive/radioactivity dissociation constant KD (~IC50)	[40]
1074910	GMEVTPSGTWLTY	N (321-333)	T-cell	HLA class I	High throughput multiplexed assay T cell binding	[2]
1087535	GMEVTPSGTWLTYTGAIK	N (321-338)	T-cell	n/d	ELISPOT IFNγ release	[50]
1143883	GMEVTPSGTWLTYTGAIKLD	N (321-340)	T-cell	HLA class I HLA class II HLA-DRB1*07:01 HLA-DRB5*01:01	binding assay qualitative binding ICS IFNγ release ICS TNFα release	[70,95]
1312574	GMEVTPSGTWLTYTG	N (321-335)	T-cell	HLA class I HLA class II	biological activity activation ELISPOT IFNγ release ELISPOT TNFα release ICS IFNγ release	[58,65,93]
41460	MEVTPSGTW	(N-322-330)	T-cell	HLA-B*44:03	biological activity degranulation ICS IFNγ release multimer/tetramer qualitative binding	[51]
190494	MEVTPSGTWL	N (322-331)	T-cell	HLA-B*40:01	biological activity activation biological activity degranulation ELISPOT IFNγ release	[5,17,47,50,51,57,67,

Epitope ID	Epitope sequence	Protein (position)	Assay type	Allele	Assay	Ref
					ICS IFNγ, TNFα<br>in vitro assay cytotoxicity<br>ICS granzyme B release<br>ICS perforin release<br>multimer/tetramer qualitative binding	68,96–98]
193441	EVTPSGTWLTY	N (323-333)	T-cell	HLA-A*26:01	biological activity activation	[5]
71461	VTPSGTWLTY	N (324-333	T-cell	HLA-A*30:02	biological activity activation	[5]
			MHC	HLA-A*30:02<br>HLA-A*29:02<br>HLA-A*26:01<br>HLA-A*01:01	purified MHC/competitive/radioactivity dissociation constant KD (~IC50)	[6]
65763	TPSGTWLTY	N (325-333)	T-cell	HLA-B*35:01	biological activity activation	[5,67]
			MHC	HLA-A*29:02<br>HLA-A*11:01	purified MHC/direct/fluorescence dissociation constant KD (~EC50)	[6,99]
1313778	TPSGTWLTYTGAIKL	N (325-339)	T-cell	HLA class I	ELISPOT IFNγ release	[58]
1310726	PSGTWLTYTGAIKLD	N (326-340)	T-cell	HLA-DRB1*01:02<br>HLA-DQB1*06:03<br>HLA-DQB1*06:03<br>HLA-DRB1*15:01<br>HLA class II	biological activity activation<br>ELISPOT IFNγ release<br>ELISPOT IL-5 release<br>ICS IFNγ release	[5,13,65]
			B-cell		ELISA qualitative binding	[21]
			MHC	HLA-DRB1*15:01<br>HLA-DRB5*01:01<br>HLA-DRB3*01:01<br>HLA-DRB1*07:01<br>HLA-DRB1*03:01<br>HLA-DRB1*11:01<br>HLA-DRB1*08:02<br>HLA-DRB1*04:01<br>HLA-DRB1*12:01<br>HLA-DRB1*04:05	purified MHC/competitive/radioactivity half maximal inhibitory concentration (IC50)	[5]
1310464	GTWLTYTGAIKLDDK	N (328-342)	T-cell	HLA-DR	biological activity activation<br>ELISPOT IFNγ release<br>ICS IFNγ, TNFα release	[52,57]
1087763	TWLTYTGAIKLDDKDPNF	N (329-346)	T-cell	HLA class II	ELISPOT IFNγ release	[50]
1313817	TWLTYTGAIKLDDKD	N (329-343)	T-cell	HLA class II	ELISA IFNγ release	[11]
1707900	WLTYTGAIKL	N (330-339)	T-cell	HLA class II<br>HLA-A*02:01	ICS IFNγ release	[8]
1321430	LTYTGAIKLDDKDPN	N (331-345)	T-cell	HLA class II<br>HLA-DRB1*07:01	ICS IFNγ release<br>biological activity activation	[5,65]
1314052	YTGAIKLDDKDPNFK	N (333-347)	T-cell	HLA class II	ICS IFNγ release	[8]
1314325	AIKLDDKDPNFKDQV	N (336-350)	T-cell	HLA class II<br>HLA-DRB1*03:01	ICS IFNγ release<br>biological activity activation	[5,65]
1312709	IKLDDKDPNFKDQVI	N (337-351)	T-cell	HLA class I, II	biological activity activation<br>ELISPOT IFNγ release	[58]
1125078	KLDDKDPNF	N (338-346)	T-cell	HLA-A*02:01	ELISPOT IFNγ release<br>multimer/tetramer qualitative binding	[30,41]
1312308	DKDPNFKDQVILLNK	N (341-355)	T-cell	n/d	ELISPOT IFNγ release	[65]
1309135	PNFKDQVILLNKHIDAYK	N (344-361)	T-cell	HLA class II	ELISPOT IFNgγ release	[92]
1479739	NFKDQVILLNKHIDAYKTF	N (345-363)	T-cell	HLA class II	ELISA IFNγ release	[11]
1310398	FKDQVILLNKHIDAY	N (346-360)	T-cell	HLA-DRB1*16:02	biological activity activation	[5,65]

Epitope ID	Epitope sequence	Protein (position)	Assay type	Allele	Assay	Ref
				HLA-DRB1*15:01<br>HLA-DRB1*14:06<br>HLA-DRB1*14:01<br>HLA class II<br>HLA-DRB1*03:01<br>HLA class II<br>HLA-DRB1*03:01<br>HLA-DRB1*04:01<br>HLA-DRB1*09:01<br>HLA-DRB1*11:01<br>HLA-DRB1*12:01<br>HLA-DRB3*01:01<br>HLA-DRB4*01:01<br>HLA-DRB5*01:01<br>HLA-DRB1*16:02<br>HLA-DRB1*15:01<br>HLA-DQB1*06:02<br>HLA-DRB1*14:01<br>HLA-DPB1*02:01<br>HLA-DPA1*01:03/DPB1*04:01<br>HLA-DQA1*01:02/DQB1*06:02<br>HLA-DRB1*15:01<br>HLA-DQA1*01:01/DQB1*05:01<br>HLA-DRB1*01:01<br>HLA-DRB1*07:01<br>HLA-DQA1*03:01/DQB1*03:02<br>HLA-DRB1*13:02<br>HLA-DQA1*05:01/DQB1*02:01<br>HLA-DRB1*04:05<br>HLA-DRB3*02:02<br>HLA-DRB1*08:02<br>HLA-DQA1*05:01/DQB1*03:01<br>HLA-DRB1*14:06<br>HLA class II	ICS IFNγ release	
				HLA-DPA1*01:03/DPB1*04:01<br>HLA-DRB1*13:02<br>HLA-DRB1*01:01<br>HLA-DRB1*08:02<br>HLA-DRB1*01:01<br>HLA-DRB5*01:01<br>HLA-DRB1*15:01<br>HLA-DRB3*01:01<br>HLA-DRB3*02:02	Purified MHC/competitive/radioactivity half maximal inhibitory concentration (IC50)	[5,65]

Epitope ID	Epitope sequence	Protein (position)	Assay type	Allele	Assay	Ref
				HLA-DRB1*15:01<br>HLA-DRB1*11:01<br>HLA-DRB1*07:01<br>HLA-DRB1*12:01<br>HLA-DPB1*02:01<br>HLA-DRB4*01:01<br>HLA-DRB1*08:02<br>HLA-DQA1*01:02/DQB1*06:02<br>HLA-DQA1*01:01/DQB1*05:01<br>HLA-DRB1*09:01<br>HLA-DRB1*04:05<br>HLA-DQA1*03:01/DQB1*03:02<br>HLA-DRB1*07:01<br>HLA-DRB1*04:05<br>HLA-DRB1*11:01<br>HLA-DRB1*12:01<br>HLA-DRB1*03:01<br>HLA-DQA1*05:01/DQB1*02:01<br>HLA-DRB1*04:01<br>HLA-DQA1*05:01/DQB1*03:01<br>HLA-DRB3*01:01<br>HLA-DRB5*01:01<br>HLA-DRB1*03:01		
1313394	QVILLNKHIDAYKTF	N (349-363)	T-cell	HLA class I, II	biological activity activation<br>ELISA IFNγ release	[11,58]
1860344	VILLNKHIDAYKTFPPTEPKKDKKK	N (350-374)	T-cell	HLA class I, II	biological activity activation<br>ELISPOT IFNγ release	[69]
27182	ILLNKHIDA	N (351-359)	T-cell	HLA class I<br>HLA-A*02:01	ELISPOT IFNγ release<br>High throughput multiplexed assay T cell binding<br>ICS IFNγ, TNFα release<br>multimer/tetramer qualitative binding	[2,28,77,78]
27183	ILLNKHIDAYKTFPP	N (351-365)	T-cell	HLA-DRB1*14:06<br>HLA-DRB1*15:01<br>HLA class II	biological activity activation<br>ELISPOT IFNγ release<br>ICS IFNγ release	[5,65]
			MHC	HLA-DRB1*15:01<br>HLA-DRB1*01:01<br>HLA-DRB1*11:01<br>HLA-DRB1*04:05<br>HLA-DRB3*01:01<br>HLA-DRB1*07:01<br>HLA-DRB1*08:02<br>HLA-DRB5*01:01	purified MHC/competitive/radioactivity half maximal inhibitory concentration (IC50)	[5]
125100	ILLNKHID	N (351-358)	T-cell	HLA-A*02:01	ELISPOT IFNγ release<br>ICS IFNγ, TNFα release	[77,78]

Epitope ID	Epitope sequence	Protein (position)	Assay type	Allele	Assay	Ref
37611	LLNKHIDAYKTFPPTEPK	N (352-369)	T-cell	HLA-A*03:01<br>HLA-A*11:01<br>+ n/d	ELISPOT IFNγ release	[50,92,100,101]
1310599	LLNKHIDAY	N (352-360)	T-cell	HLA-B*15:01	multimer/tetramer qualitative binding	[4]
1310109	HIDAYKTFPPTEPKK	N (356-370)	T-cell	HLA class II	biological activity activation<br>ICS IFNγ release	[5,65]
			B-cell		ELISA qualitative binding	[21]
1074864	AYKTFPPTEPK	N (359-369)	T-cell		High throughput multiplexed assay<br>T cell binding	[2]
1328121	YKTFPPTEPK	N (360-369)	T-cell	HLA-A*68:01<br>HLA-A*11:01<br>HLA class II	biological activity activation<br>ICS IFNγ, TNFα release<br>in vitro assay cytotoxicity<br>multimer/tetramer qualitative binding	[5,8]
33667	KTFPPTEPK	N (361-369)	T-cell	HLA-A*03:01<br>HLA-A*31:01<br>HLA-A*11:01<br>HLA-A*68:01	biological activity activation<br>ELISA IFNγ release<br>ELISA IL-2 release<br>ELISA TNFα release<br>ICS IFNγ release	[4,5,30,43,50,53,55,96,102]
			MHC	HLA-A*03:01<br>HLA-A*31:01<br>HLA-A*11:01<br>HLA-A*68:01	cellular MHC/mass spectrometry ligand presentation<br>purified MHC/competitive/fluorescence qualitative binding<br>purified MHC/competitive/radioactivitydissociation constant KD (~IC50)<br>purified MHC qualitative binding	[6,44,45,75,99,102–104]
33668	KTFPPTEPKK	N (361-370)	T-cell	HLA-A*03:01<br>HLA-A*11:01	biological activity activation<br>ELISPOT IFNγ release<br>ICS IFNγ release	[5,17,57]
			MHC	HLA-A*03:01<br>HLA-A*31:01<br>HLA-A*11:01<br>HLA-A*68:01	purified MHC/competitive/radioactivitydissociation constant KD (~IC50)	[6]
33669	KTFPPTEPKKDKKKK	N (361-375)	T-cell	HLA class II	ICS IFNγ release	[65]
			B-cell		biological activity agglutination<br>ELISA qualitative binding<br>Microarray qualitative binding	[79,105]
1319977	KTFPPTEPKKDKKK	N (361-374)	T-cell	HLA-A*03:01	biological activity activation	[5]
1548775	KIFPPTEPK	N (361-369)	T-cell	HLA-A*11:01	ELISA IFNγ release	[30]
1549045	KTFPPIEPK	N (361-369)	T-cell	HLA-A*11:01	ELISA IFNγ release	[30]
1549049	KTLPPTEPK	N (361-369)	T-cell	HLA-A*11:01	ELISA IFNγ release	[30]

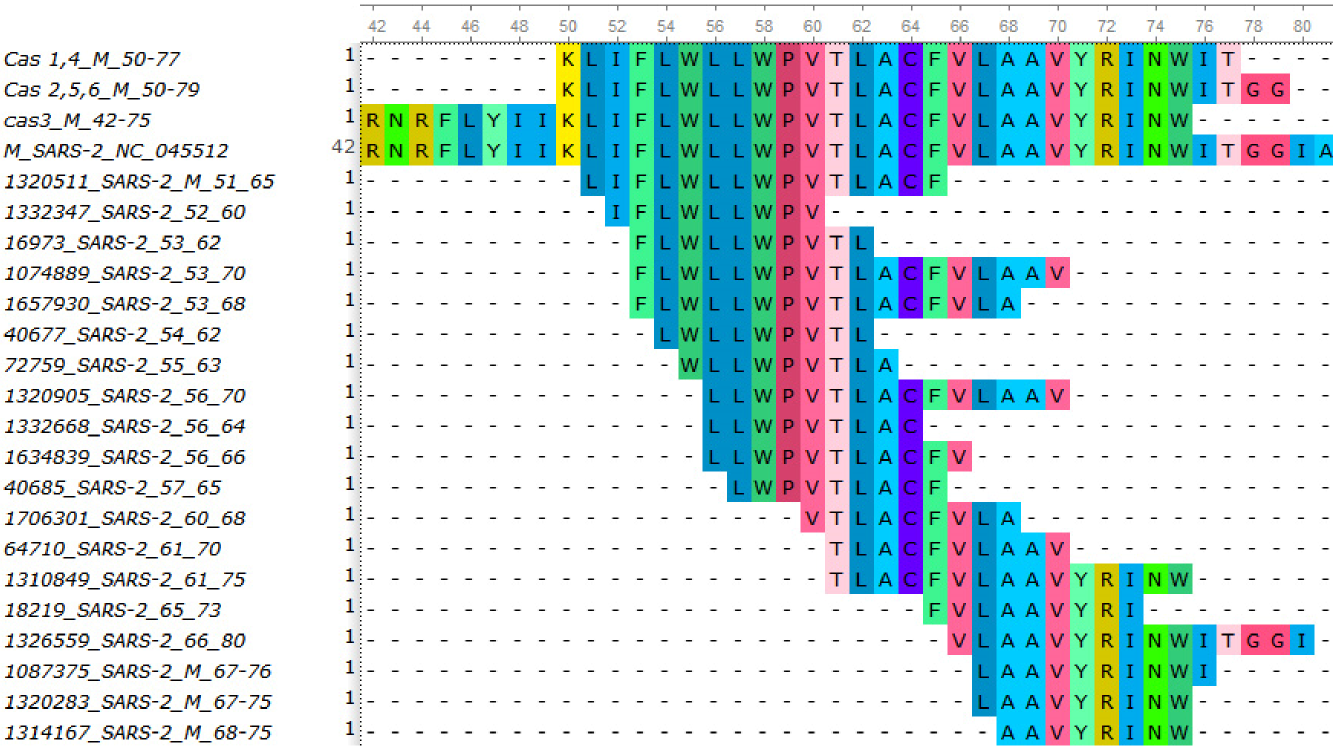

Figure S8. 42-79 fragments of membrane protein aligned with peptides established as immunogenic/MHC molecule binding epitopes in experimental studies. This fragment is a part of cassettes #1-6. Epitopes' IDs in Immune Epitope Database are indicated on the left. References and additional information regarding these epitopes are listed in Table S10. This fragment corresponds to transmembrane helix 2 and linker to 3rd helix [106].

Table S10. Epitopes in 42-79 fragment of membrane protein, deposited in IEDB, additional information and references

Epitope ID	Epitope sequence	Protein (position)	Assay type	Allele	Assay	Ref
1320511	LIFLWLLWPV TLACF	M (51-65)	T-cell	HLA-DQB1*05:01<br>HLA-DRB1*01:02<br>HLA-DQB1*05:03<br>HLA class II	biological activity activation<br>ICS IFNγ release	[5,65]
1332347	IFLWLLWPV	M (52-60)	T-cell	HLA-A*02:01	ELISPOT IFNg release	[15]
16973	FLWLLWPVT L	M (53-62)	T-cell	HLA-A*02:01	multimer/tetramer qualitative binding	[4,31]
			T-cell	HLA-A*02:01<br>HLA-A*02:02<br>HLA-A*02:03<br>HLA-A*02:06	cellular MHC/direct/fluorescence qualitative binding<br>purified MHC/competitive/radioactivity dissociation constant KD (~IC50)	[6,32,107]
1074889	FLWLLWPVT LACFVLAAV	M (53-70)	T-cell	HLA class I	High throughput multiplexed assay T cell binding	[5]
1657930	FLWLLWPVT LACFVLA	M (53-68)	T-cell	HLA class I, II	biological activity, activation	[22]
40677	LWLLWPVTL	M (54-62)	T-cell	HLA-A*24:02	ICS IFNγ release	[54]
			MHC	HLA-A*23:01<br>HLA-A*24:02<br>HLA-A*29:02	purified MHC/competitive/radioactivity dissociation constant KD (~IC50)	[6]
72759	WLLWPVTLA	M (55-63)	T-cell	HLA-A*02:01	ICS IFNγ release	[29]
			MHC	HLA-A*02:01<br>HLA-A*02:02<br>HLA-A*02:03<br>HLA-A*02:06<br>HLA-A*68:02	purified MHC/competitive/radioactivity half maximal inhibitory concentration (IC50)	[6,32]

Epitope ID	Epitope sequence	Protein (position)	Assay type	Allele	Assay	Ref
1320905	LLWPVTLACF VLAAV	M (56-70)	T-cell	HLA-DQB1*05:03<br>HLA-DQB1*06:03<br>HLA-DQB1*05:01<br>HLA class II	biological activity activation<br>ICS IFNγ release	[5,65]
1332668	LLWPVTLAC	M (56-64)	T-cell	HLA-A*02:01	multimer/tetramer qualitative binding	[4]
1634839	LLWPVTLACF V	M (56-66)	T-cell	HLA-A*02:01	multimer/tetramer qualitative binding	[31]
40685	LWPVTLACF	M (57-65)	T-cell	HLA-A*24:02	ICS IFNγ release	[8,54]
1706301	VTLACFVLA	M (60-68)	T-cell	HLA-A*02:06<br>HLA class II	CFSE proliferation<br>ICS IFNγ release	[8]
64710	TLACFVLAAV	M (61-70)	T-cell	HLA-A*02:01<br>HLA-A*02:07<br>HLA class I	CFSE proliferation<br>ELISPOT IFNγ release<br>ICS IFNγ release<br>multimer/tetramer qualitative binding	[8,27,28, 62,107]
			MHC	HLA-A*02:01<br>HLA-A*02:02<br>HLA-A*02:03<br>HLA-A*02:06<br>HLA-A*02:07<br>HLA-A*68:02	purified MHC qualitative binding purified MHC/competitive/radioactivity half maximal inhibitory concentration (IC50)<br>purified MHC/direct/fluorescence 50% dissociation temperature<br>cellular MHC/competitive/fluorescence qualitative binding	[6,8,27,32,47,107]
1310849	TLACFVLAAV YRINW	M (61-75)	T-cell	n/d	ELISPOT IFNγ release	[65]
			B-cell		ELISA qualitative binding	[21]
18219	FVLAAVYRI	M (65-73)	T-cell	HLA-A*02:01<br>HLA-A*68:02	51 chromium cytotoxicity<br>biological activity activation<br>ICS IFNγ, TNFα release<br>multimer/tetramer qualitative binding	[4,5,8,27, 31]
			MHC	HLA-A*02:01<br>HLA-A*02:02<br>HLA-A*02:03<br>HLA-A*02:06<br>HLA-A*02:07<br>HLA-A*68:02	purified MHC qualitative binding<br>purified MHC/competitive/radioactivity half maximal inhibitory concentration (IC50)<br>purified MHC/direct/fluorescence 50% dissociation temperature<br>cellular MHC/competitive/fluorescence qualitative binding	[6,8,27,31,32]
1326559	VLAAVYRIN WITGGI	M (66-80)	T-cell	HLA-DRB1*12:02<br>HLA-DQB1*05:03<br>HLA-DRB1*14:01<br>HLA-DQB1*05:01<br>HLA class II	biological activity activation<br>ICS IFNγ release	[5,65]
			MHC	HLA-DRB1*15:01<br>HLA-DRB1*09:01<br>HLA-DRB5*01:01<br>HLA-DRB1*11:01<br>HLA-DRB3*02:02<br>HLA-DRB1*07:01<br>HLA-DRB1*04:05<br>HLA-DRB1*08:02<br>HLA-DRB4*01:01<br>HLA-DRB1*01:01<br>HLA-DRB1*04:01<br>HLA-DRB1*13:02	purified MHC/competitive/radioactivity half maximal inhibitory concentration (IC50)	[5]
1087375	LAAVYRINWI	M (67-76)	T-cell	HLA class I	High throughput multiplexed assay T cell binding	[2]
1320283	LAAVYRINW	M (67-75)	T-cell	HLA-B*57:01	biological activity activation	[5]
1314167	AAVYRINW	M (68-75)	T-cell	HLA-B*57:01	biological activity activation	[5]

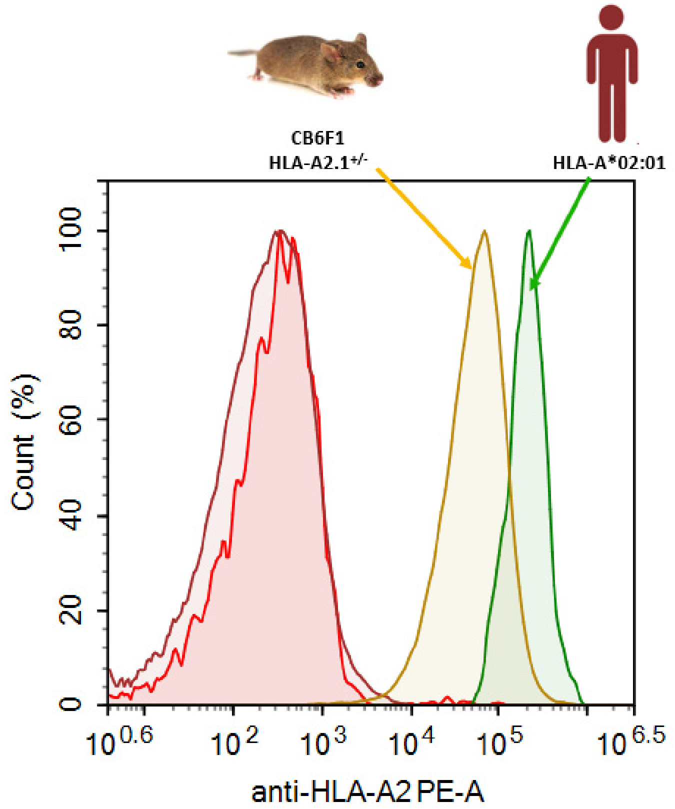

Figure S9. Expression of the HLA-A2 molecules on the mouse splenic cells and HLA-A*02:01 positive donor as detected using the PE Anti-HLA A2 antibody [BB7.2]. Whole blood was stained with antibody and the cells were processed on a flow cytometer.

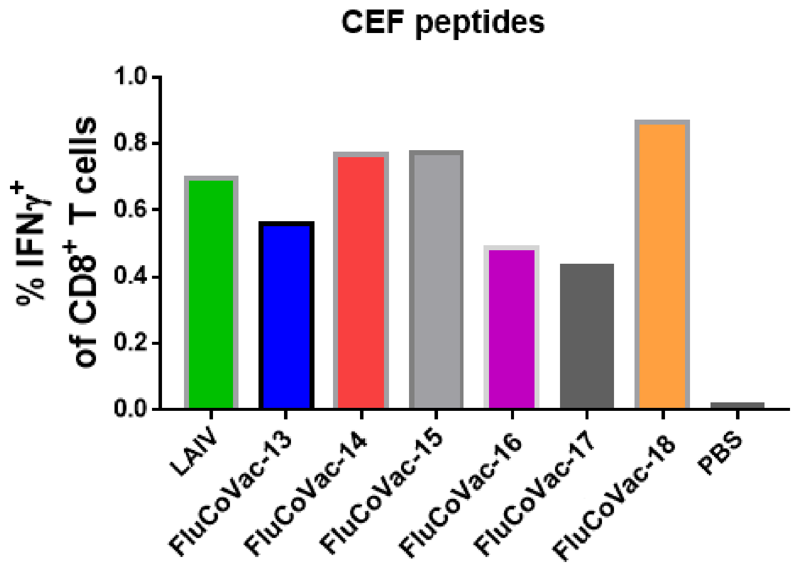

Figure S10. Levels of IFNγ-producing cytotoxic T cells after stimulation of mouse splenocytes with CEF peptide mix. Mice were immunized twice with 10^6 EID_{50} of each test virus within a 3-week interval. On day 10 post second dose splenocytes (n=7) were collected and CEF-specific CTL response was assessed by ICS assay.

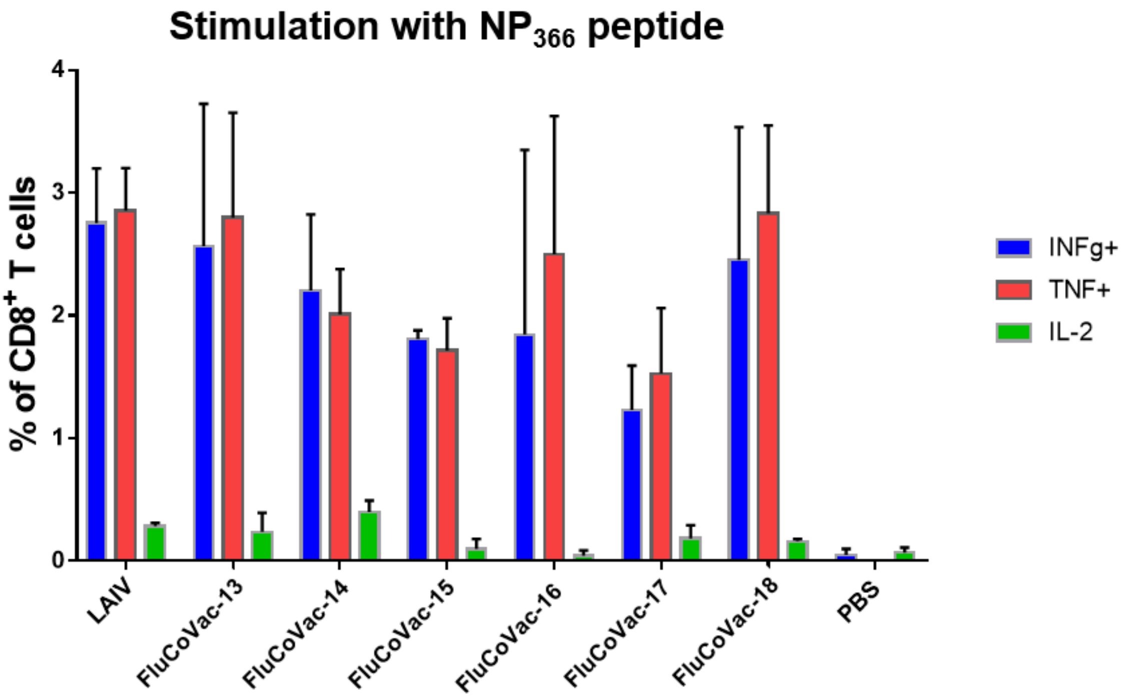

Figure S11. Levels of cytokine-producing cytotoxic T cells after stimulation of mouse splenocytes with influenza NP_{366} peptide. Mice were immunized twice with 10^6 EID_{50} of each test virus within a 3-week interval. On day 10 post second dose splenocytes (n=7) were collected and NP_{366}-specific CTL response was assessed by ICS assay.

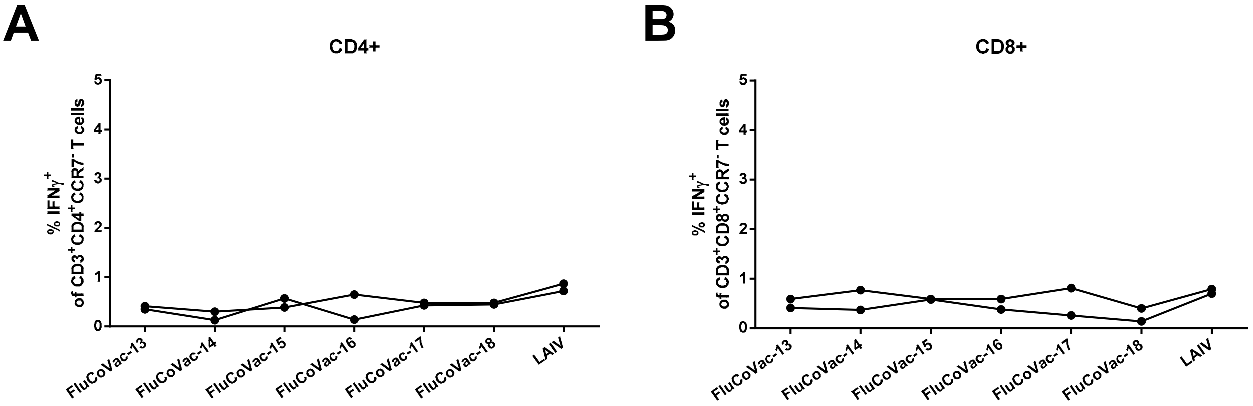

Figure S12. The levels of IFNγ-producing helper (A) and effector memory (B) T cell subsets in PBMC samples of naïve individuals stimulated in vitro with the studied recombinant LAIV/SARS-CoV-2 viruses.

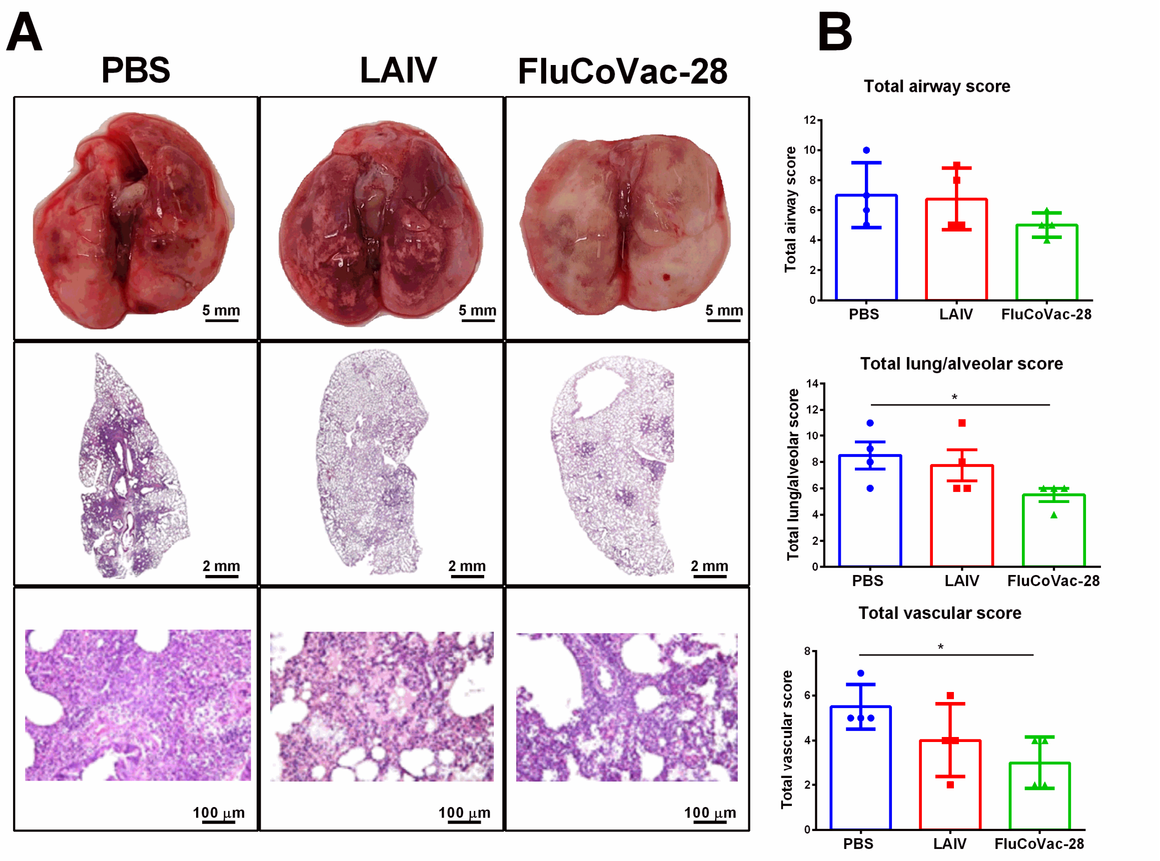

Figure S13. Histopathological evaluation of lung tissues of immunized Syrian hamsters on day 5 after challenge with SARS-CoV-2 Wuhan virus. **A.** Representative micrographs of the lungs and lung sections stained with Hematoxylin & Eosin. **B.** Semi-quantitative analyses of the changes in airway, lung/alveolar and vascular systems. Data were analyzed by one-way ANOVA with Tukey's post-hoc multiple analyses test. *—$p < 0.05$.

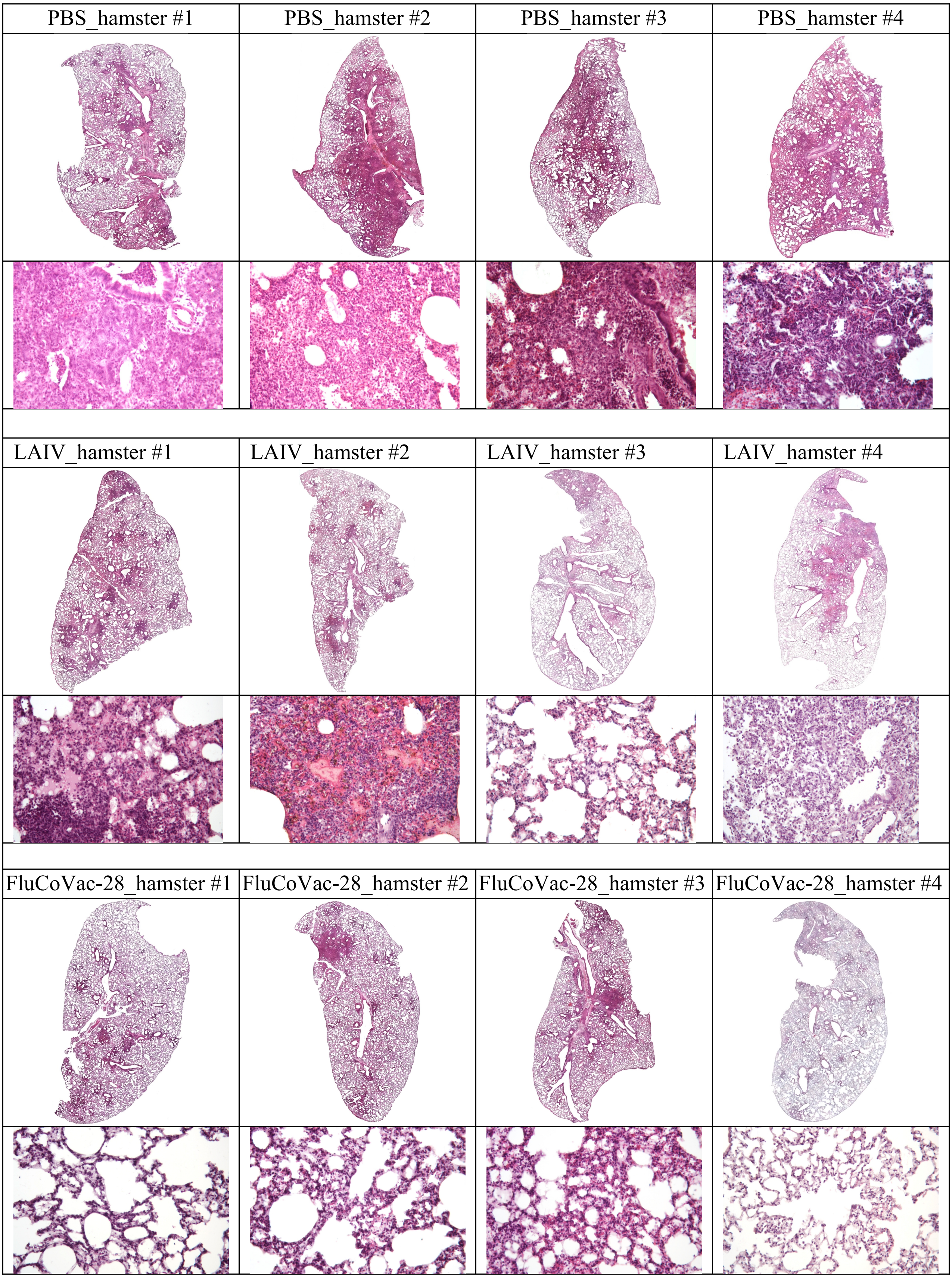

Figure S14. Overview of histopathological changes in lung tissues of immunized Syrian hamsters on day 5 after challenge with SARS-CoV-2 Delta variant. Representative micrographs of the lung sections stained with Hematoxylin & Eosin of all four animals in each test group. Lung sections are shown at magnifications of 50× (upper figure) and 200× (lower figure) for each hamster.

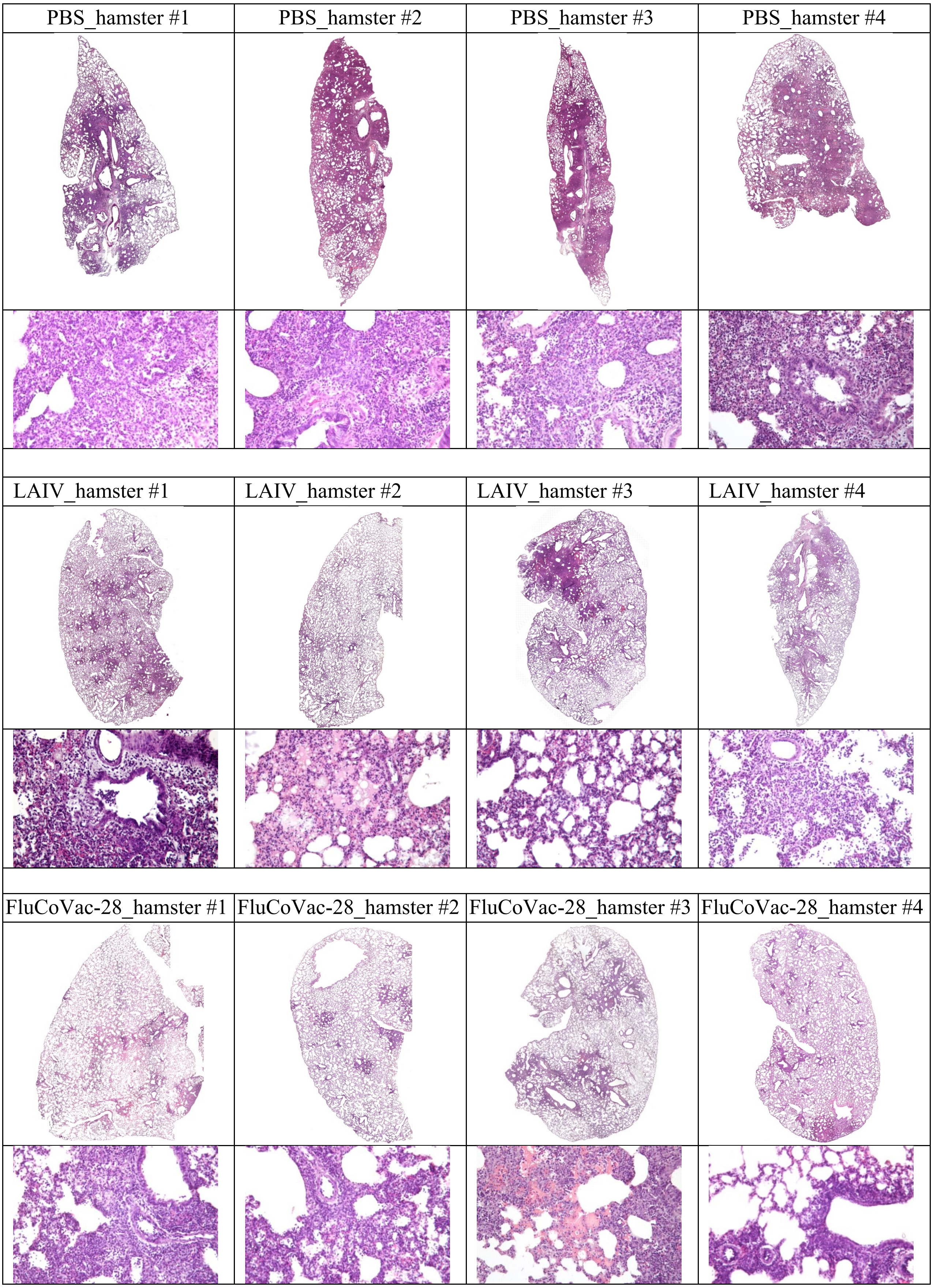

Figure S15. Overview of histopathological changes in lung tissues of immunized Syrian hamsters on day 5 after challenge with SARS-CoV-2 Wuhan variant. Representative micrographs of the lung sections stained with Hematoxylin & Eosin of all four animals in each test group. Lung sections are shown at magnifications of 50× (upper figure) and 200× (lower figure) for each hamster.

References

1. Grifoni, A.; Sidney, J.; Zhang, Y.; Scheuermann, R.H.; Peters, B.; Sette, A. A Sequence Homology and Bioinformatic Approach Can Predict Candidate Targets for Immune Responses to SARS-CoV-2. *Cell Host Microbe* **2020**, *27*, 671–680.e2. https://doi.org/10.1016/j.chom.2020.03.002.
2. Snyder, T.M.; Gittelman, R.M.; Klinger, M.; May, D.H.; Osborne, E.J.; Taniguchi, R.; Zahid, H.J.; Kaplan, I.M.; Dines, J.N.; Noakes, M.T.; et al. Magnitude and Dynamics of the T-Cell Response to SARS-CoV-2 Infection at Both Individual and Population Levels. *medRxiv* **2020**. https://doi.org/10.1101/2020.07.31.20165647.
3. Mishra, N.; Huang, X.; Joshi, S.; Guo, C.; Ng, J.; Thakkar, R.; Wu, Y.; Dong, X.; Li, Q.; Pinapati, R.S.; et al. Immunoreactive Peptide Maps of SARS-CoV-2. *Commun. Biol.* **2021**, *4*, 225. https://doi.org/10.1038/s42003-021-01743-9.
4. Saini, S.K.; Hersby, D.S.; Tamhane, T.; Povlsen, H.R.; Amaya Hernandez, S.P.; Nielsen, M.; Gang, A.O.; Hadrup, S.R. SARS-CoV-2 Genome-Wide T Cell Epitope Mapping Reveals Immunodominance and Substantial $CD8^+$ T Cell Activation in COVID-19 Patients. *Sci. Immunol.* **2021**, *6*, eabf7550. https://doi.org/10.1126/sciimmunol.abf7550.
5. Tarke, A.; Sidney, J.; Kidd, C.K.; Dan, J.M.; Ramirez, S.I.; Yu, E.D.; Mateus, J.; da Silva Antunes, R.; Moore, E.; Rubiro, P.; et al. Comprehensive Analysis of T Cell Immunodominance and Immunoprevalence of SARS-CoV-2 Epitopes in COVID-19 Cases. *Cell Rep. Med.* **2021**, *2*, 100204. https://doi.org/10.1016/j.xcrm.2021.100204.
6. Sidney, J.; Botten, J.; Neuman, B.; Buchmeier, M.; Sette, A. Large Scale Analysis of Peptide-HLA Class I Interactions (Direct IEDB Submissions 1000402, 1000425). 2006. Available online: http://www.iedb.org/reference/1000425 (accessed on 25 February 2020).
7. Mahajan, S.; Kode, V.; Bhojak, K.; Karunakaran, C.; Lee, K.; Manoharan, M.; Ramesh, A.; Hv, S.; Srivastava, A.; Sathian, R.; et al. Immunodominant T-Cell Epitopes from the SARS-CoV-2 Spike Antigen Reveal Robust Pre-Existing T-Cell Immunity in Unexposed Individuals. *Sci. Rep.* **2021**, *11*, 13164. https://doi.org/10.1038/s41598-021-92521-4.
8. Jin, X.; Ding, Y.; Sun, S.; Wang, X.; Zhou, Z.; Liu, X.; Li, M.; Chen, X.; Shen, A.; Wu, Y.; et al. Screening HLA-A-Restricted T Cell Epitopes of SARS-CoV-2 and the Induction of CD8+ T Cell Responses in HLA-A Transgenic Mice. *Cell. Mol. Immunol.* **2021**, *18*, 2588–2608. https://doi.org/10.1038/s41423-021-00784-8.
9. Low, J.S.; Vaqueirinho, D.; Mele, F.; Foglierini, M.; Jerak, J.; Perotti, M.; Jarrossay, D.; Jovic, S.; Perez, L.; Cacciatore, R.; et al. Clonal Analysis of Immunodominance and Cross-Reactivity of the CD4 T Cell Response to SARS-CoV-2. *Science* **2021**, *372*, 1336–1341. https://doi.org/10.1126/science.abg8985.
10. Zhang, J.; Xiao, T.; Cai, Y.; Chen, B. Structure of SARS-CoV-2 Spike Protein. *Curr. Opin. Virol.* **2021**, *50*, 173–182. https://doi.org/10.1016/j.coviro.2021.08.010.
11. Verhagen, J.; van der Meijden, E.D.; Lang, V.; Kremer, A.E.; Völkl, S.; Mackensen, A.; Aigner, M.; Kremer, A.N. Human CD4+ T Cells Specific for Dominant Epitopes of SARS-CoV-2 Spike and Nucleocapsid Proteins with Therapeutic Potential. *Clin. Exp. Immunol.* **2021**, *205*, 363–378. https://doi.org/10.1111/cei.13627.
12. Knierman, M.D.; Lannan, M.B.; Spindler, L.J.; McMillian, C.L.; Konrad, R.J.; Siegel, R.W. The Human Leukocyte Antigen Class II Immunopeptidome of the SARS-CoV-2 Spike Glycoprotein. *Cell Rep.* **2020**, *33*, 108454. https://doi.org/10.1016/j.celrep.2020.108454.
13. Mateus, J.; Grifoni, A.; Tarke, A.; Sidney, J.; Ramirez, S.I.; Dan, J.M.; Burger, Z.C.; Rawlings, S.A.; Smith, D.M.; Phillips, E.; et al. Selective and Cross-Reactive SARS-CoV-2 T Cell Epitopes in Unexposed Humans. *Science* **2020**, *370*, 89–94. https://doi.org/10.1126/science.abd3871.
14. Loyal, L.; Braun, J.; Henze, L.; Kruse, B.; Dingeldey, M.; Reimer, U.; Kern, F.; Schwarz, T.; Mangold, M.; Unger, C.; et al. Cross-Reactive CD4+ T Cells Enhance SARS-CoV-2 Immune Responses upon Infection and Vaccination. *Science* **2021**, *374*, eabh1823. https://doi.org/10.1126/science.abh1823.
15. Prakash, S.; Srivastava, R.; Coulon, P.-G.; Dhanushkodi, N.R.; Chentoufi, A.A.; Tifrea, D.F.; Edwards, R.A.; Figueroa, C.J.; Schubl, S.D.; Hsieh, L.; et al. Genome-Wide B Cell, CD4+, and CD8+ T Cell Epitopes That Are Highly Conserved between Human and Animal Coronaviruses, Identified from SARS-CoV-2 as Targets for Preemptive Pan-Coronavirus Vaccines. *J. Immunol.* **2021**, *206*, 2566–2582. https://doi.org/10.4049/jimmunol.2001438.
16. Shomuradova, A.S.; Vagida, M.S.; Sheetikov, S.A.; Zornikova, K.V.; Kiryukhin, D.; Titov, A.; Peshkova, I.O.; Khmelevskaya, A.; Dianov, D.V.; Malasheva, M.; et al. SARS-CoV-2 Epitopes Are Recognized by a Public and Diverse Repertoire of Human T Cell Receptors. *Immunity* **2020**, *53*, 1245–1257.e5. https://doi.org/10.1016/j.immuni.2020.11.004.
17. Wagner, K.I.; Mateyka, L.M.; Jarosch, S.; Grass, V.; Weber, S.; Schober, K.; Hammel, M.; Burrell, T.; Kalali, B.; Poppert, H.; et al. Recruitment of Highly Cytotoxic CD8+ T Cell Receptors in Mild SARS-CoV-2 Infection. *Cell Rep.* **2022**, *38*, 110214. https://doi.org/10.1016/j.celrep.2021.110214.
18. Lv, Y.; Ruan, Z.; Wang, L.; Ni, B.; Wu, Y. Identification of a Novel Conserved HLA-A*0201-Restricted Epitope from the Spike Protein of SARS-CoV. *BMC Immunol.* **2009**, *10*, 61. https://doi.org/10.1186/1471-2172-10-61.
19. Woldemeskel, B.A.; Garliss, C.C.; Blankson, J.N. SARS-CoV-2 MRNA Vaccines Induce Broad CD4+ T Cell Responses That Recognize SARS-CoV-2 Variants and HCoV-NL63. *J. Clin. Invest.* **2021**, *131*, 149335. https://doi.org/10.1172/JCI149335.
20. Liu, R.; Jiang, W.; Mellins, E.D. Yeast Display of MHC-II Enables Rapid Identification of Peptide Ligands from Protein Antigens (RIPPA). *Cell. Mol. Immunol.* **2021**, *18*, 1847–1860. https://doi.org/10.1038/s41423-021-00717-5.
21. Schwarz, T.; Heiss, K.; Mahendran, Y.; Casilag, F.; Kurth, F.; Sander, L.E.; Wendtner, C.-M.; Hoechstetter, M.A.; Müller, M.A.; Sekul, R.; et al. SARS-CoV-2 Proteome-Wide Analysis Revealed Significant Epitope Signatures in COVID-19 Patients. *Front. Immunol.* **2021**, *12*, 629185. https://doi.org/10.3389/fimmu.2021.629185.

22. Hu, W.; He, M.; Wang, X.; Sun, Q.; Kuang, M. Specific CD8+ TCR Repertoire Recognizing Conserved Antigens of SARS-CoV-2 in Unexposed Population: A Prerequisite for Broad-Spectrum CD8+ T Cell Immunity. *Vaccines* **2021**, *9*, 1093. https://doi.org/10.3390/vaccines9101093.
23. Quiros-Fernandez, I.; Poorebrahim, M.; Fakhr, E.; Cid-Arregui, A. Immunogenic T Cell Epitopes of SARS-CoV-2 Are Recognized by Circulating Memory and Naïve CD8 T Cells of Unexposed Individuals. *EBioMedicine* **2021**, *72*, 103610. https://doi.org/10.1016/j.ebiom.2021.103610.
24. Nathan, A.; Rossin, E.J.; Kaseke, C.; Park, R.J.; Khatri, A.; Koundakjian, D.; Urbach, J.M.; Singh, N.K.; Bashirova, A.; Tano-Menka, R.; et al. Structure-Guided T Cell Vaccine Design for SARS-CoV-2 Variants and Sarbecoviruses. *Cell* **2021**, *184*, 4401–4413.e10. https://doi.org/10.1016/j.cell.2021.06.029.
25. Wang, B.; Chen, H.; Jiang, X.; Zhang, M.; Wan, T.; Li, N.; Zhou, X.; Wu, Y.; Yang, F.; Yu, Y.; et al. Identification of an HLA-A*0201-Restricted CD8+ T-Cell Epitope SSp-1 of SARS-CoV Spike Protein. *Blood* **2004**, *104*, 200–206. https://doi.org/10.1182/blood-2003-11-4072.
26. Tsao, Y.-P.; Lin, J.-Y.; Jan, J.-T.; Leng, C.-H.; Chu, C.-C.; Yang, Y.-C.; Chen, S.-L. HLA-A*0201 T-Cell Epitopes in Severe Acute Respiratory Syndrome (SARS) Coronavirus Nucleocapsid and Spike Proteins. *Biochem. Biophys. Res. Commun.* **2006**, *344*, 63–71. https://doi.org/10.1016/j.bbrc.2006.03.152.
27. Deng, J.; Pan, J.; Qiu, M.; Mao, L.; Wang, Z.; Zhu, G.; Gao, L.; Su, J.; Hu, Y.; Luo, O.J.; et al. Identification of HLA-A2 Restricted CD8+ T Cell Epitopes in SARS-CoV-2 Structural Proteins. *J. Leukoc. Biol.* **2021**, *110*, 1171–1180. https://doi.org/10.1002/JLB.4MA0621-020R.
28. Nielsen, S.S.; Vibholm, L.K.; Monrad, I.; Olesen, R.; Frattari, G.S.; Pahus, M.H.; Højen, J.F.; Gunst, J.D.; Erikstrup, C.; Holleufer, A.; et al. SARS-CoV-2 Elicits Robust Adaptive Immune Responses Regardless of Disease Severity. *EBioMedicine* **2021**, *68*, 103410. https://doi.org/10.1016/j.ebiom.2021.103410.
29. Habel, J.R.; Nguyen, T.H.O.; van de Sandt, C.E.; Juno, J.A.; Chaurasia, P.; Wragg, K.; Koutsakos, M.; Hensen, L.; Jia, X.; Chua, B.; et al. Suboptimal SARS-CoV-2-Specific CD8+ T Cell Response Associated with the Prominent HLA-A*02:01 Phenotype. *Proc. Natl. Acad. Sci. USA* **2020**, *117*, 24384–24391. https://doi.org/10.1073/pnas.2015486117.
30. Hu, C.; Shen, M.; Han, X.; Chen, Q.; Li, L.; Chen, S.; Zhang, J.; Gao, F.; Wang, W.; Wang, Y.; et al. Identification of Cross-Reactive CD8+ T Cell Receptors with High Functional Avidity to a SARS-CoV-2 Immunodominant Epitope and Its Natural Mutant Variants. *Genes Dis.* **2022**, *9*, 216–229. https://doi.org/10.1016/j.gendis.2021.05.006.
31. Pan, K.; Chiu, Y.; Huang, E.; Chen, M.; Wang, J.; Lai, I.; Singh, S.; Shaw, R.M.; MacCoss, M.J.; Yee, C. Mass Spectrometric Identification of Immunogenic SARS-CoV-2 Epitopes and Cognate TCRs. *Proc. Natl. Acad. Sci. USA* **2021**, *118*, e2111815118. https://doi.org/10.1073/pnas.2111815118.
32. Ishizuka, J.; Grebe, K.; Shenderov, E.; Peters, B.; Chen, Q.; Peng, Y.; Wang, L.; Dong, T.; Pasquetto, V.; Oseroff, C.; et al. Quantitating T Cell Cross-Reactivity for Unrelated Peptide Antigens. *J. Immunol.* **2009**, *183*, 4337–4345. https://doi.org/10.4049/jimmunol.0901607.
33. Minervina, A.A.; Pogorelyy, M.V.; Kirk, A.M.; Crawford, J.C.; Allen, E.K.; Chou, C.-H.; Mettelman, R.C.; Allison, K.J.; Lin, C.-Y.; Brice, D.C.; et al. SARS-CoV-2 Antigen Exposure History Shapes Phenotypes and Specificity of Memory CD8 T Cells. *medRxiv* **2022**, *23*, 781–790. https://doi.org/10.1101/2021.07.12.21260227.
34. Li, M.; Liu, J.; Lu, R.; Zhang, Y.; Du, M.; Xing, M.; Wu, Z.; Kong, X.; Zhu, Y.; Zhou, X.; et al. Longitudinal Immune Profiling Reveals Dominant Epitopes Mediating Long-Term Humoral Immunity in COVID-19-Convalescent Individuals. *J. Allergy Clin. Immunol.* **2022**, *149*, 1225–1241. https://doi.org/10.1016/j.jaci.2022.01.005.
35. Oberhardt, V.; Luxenburger, H.; Kemming, J.; Schulien, I.; Ciminski, K.; Giese, S.; Csernalabics, B.; Lang-Meli, J.; Janowska, I.; Staniek, J.; et al. Rapid and Stable Mobilization of CD8+ T Cells by SARS-CoV-2 MRNA Vaccine. *Nature* **2021**, *597*, 268–273. https://doi.org/10.1038/s41586-021-03841-4.
36. Zhou, M.; Xu, D.; Li, X.; Li, H.; Shan, M.; Tang, J.; Wang, M.; Wang, F.-S.; Zhu, X.; Tao, H.; et al. Screening and Identification of Severe Acute Respiratory Syndrome-Associated Coronavirus-Specific CTL Epitopes. *J. Immunol.* **2006**, *177*, 2138–2145. https://doi.org/10.4049/jimmunol.177.4.2138.
37. Chen, H.; Hou, J.; Jiang, X.; Ma, S.; Meng, M.; Wang, B.; Zhang, M.; Zhang, M.; Tang, X.; Zhang, F.; et al. Response of Memory CD8+ T Cells to Severe Acute Respiratory Syndrome (SARS) Coronavirus in Recovered SARS Patients and Healthy Individuals. *J. Immunol.* **2005**, *175*, 591–598. https://doi.org/10.4049/jimmunol.175.1.591.
38. Wang, Y.-D.; Sin, W.-Y.F.; Xu, G.-B.; Yang, H.-H.; Wong, T.; Pang, X.-W.; He, X.-Y.; Zhang, H.-G.; Ng, J.N.L.; Cheng, C.-S.S.; et al. T-Cell Epitopes in Severe Acute Respiratory Syndrome (SARS) Coronavirus Spike Protein Elicit a Specific T-Cell Immune Response in Patients Who Recover from SARS. *J. Virol.* **2004**, *78*, 5612–5618. https://doi.org/10.1128/JVI.78.11.5612-5618.2004.
39. Meyers, L.M.; Gutiérrez, A.H.; Boyle, C.M.; Terry, F.; McGonnigal, B.G.; Salazar, A.; Princiotta, M.F.; Martin, W.D.; De Groot, A.S.; Moise, L. Highly Conserved, Non-Human-like, and Cross-Reactive SARS-CoV-2 T Cell Epitopes for COVID-19 Vaccine Design and Validation. *NPJ Vaccines* **2021**, *6*, 71. https://doi.org/10.1038/s41541-021-00331-6.
40. Sidney, J.; Botten, J.; Neuman, B.; Buchmeier, M.; Sette, A. HLA DRB1*01:01 Binding Capacity of Selected SARS-Derived Peptides. 2006. Available online: http://www.iedb.org/reference/1000399 (accessed on 25 February 2020).
41. Poran, A.; Harjanto, D.; Malloy, M.; Arieta, C.M.; Rothenberg, D.A.; Lenkala, D.; van Buuren, M.M.; Addona, T.A.; Rooney, M.S.; Srinivasan, L.; et al. Sequence-Based Prediction of SARS-CoV-2 Vaccine Targets Using a Mass Spectrometry-Based Bioinformatics Predictor Identifies Immunogenic T Cell Epitopes. *Genome Med.* **2020**, *12*, 70. https://doi.org/10.1186/s13073-020-00767-w.

42. Wu, D.; Kolesnikov, A.; Yin, R.; Guest, J.D.; Gowthaman, R.; Shmelev, A.; Serdyuk, Y.; Dianov, D.V.; Efimov, G.A.; Pierce, B.G.; et al. Structural Assessment of HLA-A2-Restricted SARS-CoV-2 Spike Epitopes Recognized by Public and Private T-Cell Receptors. *Nat. Commun.* **2022**, *13*, 19. https://doi.org/10.1038/s41467-021-27669-8.
43. Kared, H.; Redd, A.D.; Bloch, E.M.; Bonny, T.S.; Sumatoh, H.; Kairi, F.; Carbajo, D.; Abel, B.; Newell, E.W.; Bettinotti, M.P.; et al. SARS-CoV-2-Specific CD8+ T Cell Responses in Convalescent COVID-19 Individuals. *J. Clin. Invest.* **2021**, *131*, 145476. https://doi.org/10.1172/JCI145476.
44. Poluektov, Y.; George, M.; Daftarian, P.; Delcommenne, M.C. Assessment of SARS-CoV-2 Specific CD4(+) and CD8 (+) T Cell Responses Using MHC Class I and II Tetramers. *Vaccine* **2021**, *39*, 2110–2116. https://doi.org/10.1016/j.vaccine.2021.03.008.
45. Prachar, M.; Justesen, S.; Steen-Jensen, D.B.; Thorgrimsen, S.; Jurgons, E.; Winther, O.; Bagger, F.O. Identification and Validation of 174 COVID-19 Vaccine Candidate Epitopes Reveals Low Performance of Common Epitope Prediction Tools. *Sci. Rep.* **2020**, *10*, 20465. https://doi.org/10.1038/s41598-020-77466-4.
46. Gangaev, A.; Ketelaars, S.L.C.; Isaeva, O.I.; Patiwael, S.; Dopler, A.; Hoefakker, K.; De Biasi, S.; Gibellini, L.; Mussini, C.; Guaraldi, G.; et al. Identification and Characterization of a SARS-CoV-2 Specific CD8+ T Cell Response with Immunodominant Features. *Nat. Commun.* **2021**, *12*, 2593. https://doi.org/10.1038/s41467-021-22811-y.
47. Agerer, B.; Koblischke, M.; Gudipati, V.; Montaño-Gutierrez, L.F.; Smyth, M.; Popa, A.; Genger, J.-W.; Endler, L.; Florian, D.M.; Mühlgrabner, V.; et al. SARS-CoV-2 Mutations in MHC-I-Restricted Epitopes Evade CD8+ T Cell Responses. *Sci. Immunol.* **2021**, *6*, eabg6461. https://doi.org/10.1126/sciimmunol.abg6461.
48. Weingarten-Gabbay, S.; Klaeger, S.; Sarkizova, S.; Pearlman, L.R.; Chen, D.-Y.; Gallagher, K.M.E.; Bauer, M.R.; Taylor, H.B.; Dunn, W.A.; Tarr, C.; et al. Profiling SARS-CoV-2 HLA-I Peptidome Reveals T Cell Epitopes from out-of-Frame ORFs. *Cell* **2021**, *184*, 3962-3980.e17. https://doi.org/10.1016/j.cell.2021.05.046.
49. Shimizu, K.; Iyoda, T.; Sanpei, A.; Nakazato, H.; Okada, M.; Ueda, S.; Kato-Murayama, M.; Murayama, K.; Shirouzu, M.; Harada, N.; et al. Identification of TCR Repertoires in Functionally Competent Cytotoxic T Cells Cross-Reactive to SARS-CoV-2. *Commun. Biol.* **2021**, *4*, 1365. https://doi.org/10.1038/s42003-021-02885-6.
50. Peng, Y.; Mentzer, A.J.; Liu, G.; Yao, X.; Yin, Z.; Dong, D.; Dejnirattisai, W.; Rostron, T.; Supasa, P.; Liu, C.; et al. Broad and Strong Memory $CD4^+$ and $CD8^+$ T Cells Induced by SARS-CoV-2 in UK Convalescent Individuals Following COVID-19. *Nat. Immunol.* **2020**, *21*, 1336–1345. https://doi.org/10.1038/s41590-020-0782-6.
51. Schulien, I.; Kemming, J.; Oberhardt, V.; Wild, K.; Seidel, L.M.; Killmer, S.; Sugar; Daul, F.; Lago, M.S.; Decker, A.; et al. Characterization of Pre-Existing and Induced SARS-CoV-2-Specific $CD8^+$ T Cells. *Nat. Med.* **2021**, *27*, 78–85. https://doi.org/10.1038/s41591-020-01143-2.
52. Bilich, T.; Nelde, A.; Heitmann, J.S.; Maringer, Y.; Roerden, M.; Bauer, J.; Rieth, J.; Wacker, M.; Peter, A.; Hörber, S.; et al. T Cell and Antibody Kinetics Delineate SARS-CoV-2 Peptides Mediating Long-Term Immune Responses in COVID-19 Convalescent Individuals. *Sci. Transl. Med.* **2021**, *13*, eabf7517. https://doi.org/10.1126/scitranslmed.abf7517.
53. Ferretti, A.P.; Kula, T.; Wang, Y.; Nguyen, D.M.V.; Weinheimer, A.; Dunlap, G.S.; Xu, Q.; Nabilsi, N.; Perullo, C.R.; Cristofaro, A.W.; et al. Unbiased Screens Show CD8+ T Cells of COVID-19 Patients Recognize Shared Epitopes in SARS-CoV-2 That Largely Reside Outside the Spike Protein. *Immunity* **2020**, *53*, 1095–1107.e3. https://doi.org/10.1016/j.immuni.2020.10.006.
54. Rowntree, L.C.; Petersen, J.; Juno, J.A.; Chaurasia, P.; Wragg, K.; Koutsakos, M.; Hensen, L.; Wheatley, A.K.; Kent, S.J.; Rossjohn, J.; et al. SARS-CoV-2-Specific CD8+ T-Cell Responses and TCR Signatures in the Context of a Prominent HLA-A*24:02 Allomorph. *Immunol. Cell Biol.* **2021**, *99*, 990–1000. https://doi.org/10.1111/imcb.12482.
55. Zhang, H.; Deng, S.; Ren, L.; Zheng, P.; Hu, X.; Jin, T.; Tan, X. Profiling CD8+ T Cell Epitopes of COVID-19 Convalescents Reveals Reduced Cellular Immune Responses to SARS-CoV-2 Variants. *Cell Rep.* **2021**, *36*, 109708. https://doi.org/10.1016/j.celrep.2021.109708.
56. Nguyen, T.H.O.; Rowntree, L.C.; Petersen, J.; Chua, B.Y.; Hensen, L.; Kedzierski, L.; van de Sandt, C.E.; Chaurasia, P.; Tan, H.-X.; Habel, J.R.; et al. CD8+ T Cells Specific for an Immunodominant SARS-CoV-2 Nucleocapsid Epitope Display High Naive Precursor Frequency and TCR Promiscuity. *Immunity* **2021**, *54*, 1066–1082.e5. https://doi.org/10.1016/j.immuni.2021.04.009.
57. Nelde, A.; Bilich, T.; Heitmann, J.S.; Maringer, Y.; Salih, H.R.; Roerden, M.; Lübke, M.; Bauer, J.; Rieth, J.; Wacker, M.; et al. SARS-CoV-2-Derived Peptides Define Heterologous and COVID-19-Induced T Cell Recognition. *Nat. Immunol.* **2021**, *22*, 74–85. https://doi.org/10.1038/s41590-020-00808-x.
58. Zhao, J.; Wang, L.; Schank, M.; Dang, X.; Lu, Z.; Cao, D.; Khanal, S.; Nguyen, L.N.; Nguyen, L.N.T.; Zhang, J.; et al. SARS-CoV-2 Specific Memory T Cell Epitopes Identified in COVID-19-Recovered Subjects. *Virus Res.* **2021**, *304*, 198508. https://doi.org/10.1016/j.virusres.2021.198508.
59. Wang, Y.-D.; Chen, W.F. Detecting Specific Cytotoxic T Lymphocytes against SARS-Coronavirus with DimerX HLA-A2:Ig Fusion Protein. *Clin. Immunol.* **2004**, *113*, 151–154. https://doi.org/10.1016/j.clim.2004.07.004.
60. Kohyama, S.; Ohno, S.; Suda, T.; Taneichi, M.; Yokoyama, S.; Mori, M.; Kobayashi, A.; Hayashi, H.; Uchida, T.; Matsui, M. Efficient Induction of Cytotoxic T Lymphocytes Specific for Severe Acute Respiratory Syndrome (SARS)-Associated Coronavirus by Immunization with Surface-Linked Liposomal Peptides Derived from a Non-Structural Polyprotein 1a. *Antivir. Res.* **2009**, *84*, 168–177. https://doi.org/10.1016/j.antiviral.2009.09.004.
61. Rha, M.-S.; Jeong, H.W.; Ko, J.-H.; Choi, S.J.; Seo, I.-H.; Lee, J.S.; Sa, M.; Kim, A.R.; Joo, E.-J.; Ahn, J.Y.; et al. PD-1-Expressing SARS-CoV-2-Specific CD8+ T Cells Are Not Exhausted, but Functional in Patients with COVID-19. *Immunity* **2021**, *54*, 44–52.e3. https://doi.org/10.1016/j.immuni.2020.12.002.

62. de Castro, M.V.; Santos, K.S.; Apostolico, J.S.; Fernandes, E.R.; Almeida, R.R.; Levin, G.; Magawa, J.Y.; Nunes, J.P.S.; Bruni, M.; Yamamoto, M.M.; et al. Recurrence of COVID-19 Associated with Reduced T-Cell Responses in a Monozygotic Twin Pair. *Open Biol.* **2022**, *12*, 210240. https://doi.org/10.1098/rsob.210240.
63. Zhao, J.; Zhao, J.; Mangalam, A.K.; Channappanavar, R.; Fett, C.; Meyerholz, D.K.; Agnihothram, S.; Baric, R.S.; David, C.S.; Perlman, S. Airway Memory CD4+ T Cells Mediate Protective Immunity against Emerging Respiratory Coronaviruses. *Immunity* **2016**, *44*, 1379–1391. https://doi.org/10.1016/j.immuni.2016.05.006.
64. Kang, S.; Yang, M.; Hong, Z.; Zhang, L.; Huang, Z.; Chen, X.; He, S.; Zhou, Z.; Zhou, Z.; Chen, Q.; et al. Crystal Structure of SARS-CoV-2 Nucleocapsid Protein RNA Binding Domain Reveals Potential Unique Drug Targeting Sites. *Acta Pharm. Sin. B* **2020**, *10*, 1228–1238. https://doi.org/10.1016/j.apsb.2020.04.009.
65. Heide, J.; Schulte, S.; Kohsar, M.; Brehm, T.T.; Herrmann, M.; Karsten, H.; Marget, M.; Peine, S.; Johansson, A.M.; Sette, A.; et al. Broadly Directed SARS-CoV-2-Specific CD4+ T Cell Response Includes Frequently Detected Peptide Specificities within the Membrane and Nucleoprotein in Patients with Acute and Resolved COVID-19. *PLoS Pathog.* **2021**, *17*, e1009842. https://doi.org/10.1371/journal.ppat.1009842.
66. Lineburg, K.E.; Grant, E.J.; Swaminathan, S.; Chatzileontiadou, D.S.M.; Szeto, C.; Sloane, H.; Panikkar, A.; Raju, J.; Crooks, P.; Rehan, S.; et al. CD8+ T Cells Specific for an Immunodominant SARS-CoV-2 Nucleocapsid Epitope Cross-React with Selective Seasonal Coronaviruses. *Immunity* **2021**, *54*, 1055–1065.e5. https://doi.org/10.1016/j.immuni.2021.04.006.
67. Panikkar, A.; Lineburg, K.E.; Raju, J.; Chew, K.Y.; Ambalathingal, G.R.; Rehan, S.; Swaminathan, S.; Crooks, P.; Le Texier, L.; Beagley, L.; et al. SARS-CoV-2-Specific T Cells Generated for Adoptive Immunotherapy Are Capable of Recognizing Multiple SARS-CoV-2 Variants. *PLoS Pathog.* **2022**, *18*, e1010339. https://doi.org/10.1371/journal.ppat.1010339.
68. Swaminathan, S.; Lineburg, K.E.; Ambalathingal, G.R.; Crooks, P.; Grant, E.J.; Mohan, S.V.; Raju, J.; Panikkar, A.; Le Texier, L.; Tong, Z.W.M.; et al. Limited Recognition of Highly Conserved Regions of SARS-CoV-2. *Microbiol. Spectr.* **2022**, *10*, e0278021. https://doi.org/10.1128/spectrum.02780-21.
69. Piadel, K.; Haybatollahi, A.; Dalgleish, A.G.; Smith, P.L. Selection and T-Cell Antigenicity of Synthetic Long Peptides Derived from SARS-CoV-2. *J. Gen. Virol.* **2022**, *103*, 1698. https://doi.org/10.1099/jgv.0.001698.
70. Le Bert, N.; Tan, A.T.; Kunasegaran, K.; Tham, C.Y.L.; Hafezi, M.; Chia, A.; Chng, M.H.Y.; Lin, M.; Tan, N.; Linster, M.; et al. SARS-CoV-2-Specific T Cell Immunity in Cases of COVID-19 and SARS, and Uninfected Controls. *Nature* **2020**, *584*, 457–462. https://doi.org/10.1038/s41586-020-2550-z.
71. Schmidt, K.G.; Nganou-Makamdop, K.; Tenbusch, M.; El Kenz, B.; Maier, C.; Lapuente, D.; Überla, K.; Spriewald, B.; Bergmann, S.; Harrer, E.G.; et al. SARS-CoV-2-Seronegative Subjects Target CTL Epitopes in the SARS-CoV-2 Nucleoprotein Cross-Reactive to Common Cold Coronaviruses. *Front. Immunol.* **2021**, *12*, 627568. https://doi.org/10.3389/fimmu.2021.627568.
72. Sekine, T.; Perez-Potti, A.; Rivera-Ballesteros, O.; Strålin, K.; Gorin, J.-B.; Olsson, A.; Llewellyn-Lacey, S.; Kamal, H.; Bogdanovic, G.; Muschiol, S.; et al. Robust T Cell Immunity in Convalescent Individuals with Asymptomatic or Mild COVID-19. *Cell* **2020**, *183*, 158–168.e14. https://doi.org/10.1016/j.cell.2020.08.017.
73. Rammensee, H.-G.; Gouttefangeas, C.; Heidu, S.; Klein, R.; Preuß, B.; Walz, J.S.; Nelde, A.; Haen, S.P.; Reth, M.; Yang, J.; et al. Designing a SARS-CoV-2 T-Cell-Inducing Vaccine for High-Risk Patient Groups. *Vaccines* **2021**, *9*, 428. https://doi.org/10.3390/vaccines9050428.
74. Lee, E.; Sandgren, K.; Duette, G.; Stylianou, V.V.; Khanna, R.; Eden, J.-S.; Blyth, E.; Gottlieb, D.; Cunningham, A.L.; Palmer, S. Identification of SARS-CoV-2 Nucleocapsid and Spike T-Cell Epitopes for Assessing T-Cell Immunity. *J. Virol.* **2021**, *95*, e02002-20. https://doi.org/10.1128/JVI.02002-20.
75. Nagler, A.; Kalaora, S.; Barbolin, C.; Gangaev, A.; Ketelaars, S.L.C.; Alon, M.; Pai, J.; Benedek, G.; Yahalom-Ronen, Y.; Erez, N.; et al. Identification of Presented SARS-CoV-2 HLA Class I and HLA Class II Peptides Using HLA Peptidomics. *Cell Rep.* **2021**, *35*, 109305. https://doi.org/10.1016/j.celrep.2021.109305.
76. Cheung, Y.K.; Cheng, S.C.S.; Sin, F.W.Y.; Chan, K.T.; Xie, Y. Investigation of Immunogenic T-Cell Epitopes in SARS Virus Nucleocapsid Protein and Their Role in the Prevention and Treatment of SARS Infection. *Hong Kong Med. J.* **2008**, *14* (Suppl. 4), 27–30.
77. Cheung, Y.-K.; Cheng, S.C.-S.; Sin, F.W.-Y.; Chan, K.-T.; Xie, Y. Induction of T-Cell Response by a DNA Vaccine Encoding a Novel HLA-A*0201 Severe Acute Respiratory Syndrome Coronavirus Epitope. *Vaccine* **2007**, *25*, 6070–6077. https://doi.org/10.1016/j.vaccine.2007.05.025.
78. Szeto, C.; Chatzileontiadou, D.S.M.; Nguyen, A.T.; Sloane, H.; Lobos, C.A.; Jayasinghe, D.; Halim, H.; Smith, C.; Riboldi-Tunnicliffe, A.; Grant, E.J.; et al. The Presentation of SARS-CoV-2 Peptides by the Common HLA-A* 02:01 Molecule. *iScience* **2021**, *24*, 102096. https://doi.org/10.1016/j.isci.2021.102096.
79. Voss, C.; Esmail, S.; Liu, X.; Knauer, M.J.; Ackloo, S.; Kaneko, T.; Lowes, L.; Stogios, P.; Seitova, A.; Hutchinson, A.; et al. Epitope-Specific Antibody Responses Differentiate COVID-19 Outcomes and Variants of Concern. *JCI Insight* **2021**, *6*, 148855. https://doi.org/10.1172/jci.insight.148855.
80. Musicò, A.; Frigerio, R.; Mussida, A.; Barzon, L.; Sinigaglia, A.; Riccetti, S.; Gobbi, F.; Piubelli, C.; Bergamaschi, G.; Chiari, M.; et al. SARS-CoV-2 Epitope Mapping on Microarrays Highlights Strong Immune-Response to N Protein Region. *Vaccines* **2021**, *9*, 35. https://doi.org/10.3390/vaccines9010035.
81. Heffron, A.S.; McIlwain, S.J.; Amjadi, M.F.; Baker, D.A.; Khullar, S.; Armbrust, T.; Halfmann, P.J.; Kawaoka, Y.; Sethi, A.K.; Palmenberg, A.C.; et al. The Landscape of Antibody Binding in SARS-CoV-2 Infection. *PLoS Biol.* **2021**, *19*, e3001265. https://doi.org/10.1371/journal.pbio.3001265.

82. Yang, M.; He, S.; Chen, X.; Huang, Z.; Zhou, Z.; Zhou, Z.; Chen, Q.; Chen, S.; Kang, S. Structural Insight Into the SARS-CoV-2 Nucleocapsid Protein C-Terminal Domain Reveals a Novel Recognition Mechanism for Viral Transcriptional Regulatory Sequences. *Front. Chem.* **2021**, *8*, 624765. https://doi.org/10.3389/fchem.2020.624765.
83. Ohno, S.; Kohyama, S.; Taneichi, M.; Moriya, O.; Hayashi, H.; Oda, H.; Mori, M.; Kobayashi, A.; Akatsuka, T.; Uchida, T.; et al. Synthetic Peptides Coupled to the Surface of Liposomes Effectively Induce SARS Coronavirus-Specific Cytotoxic T Lymphocytes and Viral Clearance in HLA-A*0201 Transgenic Mice. *Vaccine* **2009**, *27*, 3912–3920. https://doi.org/10.1016/j.vaccine.2009.04.001.
84. Holenya, P.; Lange, P.J.; Reimer, U.; Woltersdorf, W.; Panterodt, T.; Glas, M.; Wasner, M.; Eckey, M.; Drosch, M.; Hollidt, J.-M.; et al. Peptide Microarray-Based Analysis of Antibody Responses to SARS-CoV-2 Identifies Unique Epitopes with Potential for Diagnostic Test Development. *Eur. J. Immunol.* **2021**, *51*, 1839–1849. https://doi.org/10.1002/eji.202049101.
85. Lee, Y.-S.; Hong, S.-H.; Park, H.-J.; Lee, H.-Y.; Hwang, J.-Y.; Kim, S.Y.; Park, J.W.; Choi, K.-S.; Seong, J.K.; Park, S.-I.; et al. Peptides Derived From S and N Proteins of Severe Acute Respiratory Syndrome Coronavirus 2 Induce T Cell Responses: A Proof of Concept for T Cell Vaccines. *Front. Microbiol.* **2021**, *12*, 732450. https://doi.org/10.3389/fmicb.2021.732450.
86. Joag, V.; Wijeyesinghe, S.; Stolley, J.M.; Quarnstrom, C.F.; Dileepan, T.; Soerens, A.G.; Sangala, J.A.; O'Flanagan, S.D.; Gavil, N.V.; Hong, S.-W.; et al. Cutting Edge: Mouse SARS-CoV-2 Epitope Reveals Infection and Vaccine-Elicited CD8 T Cell Responses. *J. Immunol.* **2021**, *206*, 931–935. https://doi.org/10.4049/jimmunol.2001400.
87. Brunk, F.; Moritz, A.; Nelde, A.; Bilich, T.; Casadei, N.; Fraschka, S.A.K.; Heitmann, J.S.; Hörber, S.; Peter, A.; Rammensee, H.-G.; et al. SARS-CoV-2-Reactive T-Cell Receptors Isolated from Convalescent COVID-19 Patients Confer Potent T-Cell Effector Function. *Eur. J. Immunol.* **2021**, *51*, 2651–2664. https://doi.org/10.1002/eji.202149290.
88. Heitmann, J.S.; Bilich, T.; Tandler, C.; Nelde, A.; Maringer, Y.; Marconato, M.; Reusch, J.; Jäger, S.; Denk, M.; Richter, M.; et al. A COVID-19 Peptide Vaccine for the Induction of SARS-CoV-2 T Cell Immunity. *Nature* **2022**, *601*, 617–622. https://doi.org/10.1038/s41586-021-04232-5.
89. Janice Oh, H.-L.; Ken-En Gan, S.; Bertoletti, A.; Tan, Y.-J. Understanding the T Cell Immune Response in SARS Coronavirus Infection. *Emerg. Microbes. Infect.* **2012**, *1*, e23. https://doi.org/10.1038/emi.2012.26.
90. Liu, S.-J.; Leng, C.-H.; Lien, S.-P.; Chi, H.-Y.; Huang, C.-Y.; Lin, C.-L.; Lian, W.-C.; Chen, C.-J.; Hsieh, S.-L.; Chong, P. Immunological Characterizations of the Nucleocapsid Protein Based SARS Vaccine Candidates. *Vaccine* **2006**, *24*, 3100–3108. https://doi.org/10.1016/j.vaccine.2006.01.058.
91. Somogyi, E.; Csiszovszki, Z.; Molnár, L.; Lőrincz, O.; Tóth, J.; Pattijn, S.; Schockaert, J.; Mazy, A.; Miklós, I.; Pántya, K.; et al. A Peptide Vaccine Candidate Tailored to Individuals' Genetics Mimics the Multi-Targeted T Cell Immunity of COVID-19 Convalescent Subjects. *Front. Genet.* **2021**, *12*, 684152. https://doi.org/10.3389/fgene.2021.684152.
92. Peng, H.; Yang, L.; Wang, L.; Li, J.; Huang, J.; Lu, Z.; Koup, R.A.; Bailer, R.T.; Wu, C. Long-Lived Memory T Lymphocyte Responses against SARS Coronavirus Nucleocapsid Protein in SARS-Recovered Patients. *Virology* **2006**, *351*, 466–475. https://doi.org/10.1016/j.virol.2006.03.036.
93. Keller, M.D.; Harris, K.M.; Jensen-Wachspress, M.A.; Kankate, V.V.; Lang, H.; Lazarski, C.A.; Durkee-Shock, J.; Lee, P.-H.; Chaudhry, K.; Webber, K.; et al. SARS-CoV-2–Specific T Cells Are Rapidly Expanded for Therapeutic Use and Target Conserved Regions of the Membrane Protein. *Blood* **2020**, *136*, 2905–2917. https://doi.org/10.1182/blood.2020008488.
94. Hu, H.; Li, L.; Kao, R.Y.; Kou, B.; Wang, Z.; Zhang, L.; Zhang, H.; Hao, Z.; Tsui, W.H.; Ni, A.; et al. Screening and Identification of Linear B-Cell Epitopes and Entry-Blocking Peptide of Severe Acute Respiratory Syndrome (SARS)-Associated Coronavirus Using Synthetic Overlapping Peptide Library. *J. Comb. Chem.* **2005**, *7*, 648–656. https://doi.org/10.1021/cc0500607.
95. Johansson, A.M.; Malhotra, U.; Kim, Y.G.; Gomez, R.; Krist, M.P.; Wald, A.; Koelle, D.M.; Kwok, W.W. Cross-Reactive and Mono-Reactive SARS-CoV-2 CD4+ T Cells in Prepandemic and COVID-19 Convalescent Individuals. *PLoS Pathog.* **2021**, *17*, e1010203. https://doi.org/10.1371/journal.ppat.1010203.
96. de Silva, T.I.; Liu, G.; Lindsey, B.B.; Dong, D.; Moore, S.C.; Hsu, N.S.; Shah, D.; Wellington, D.; Mentzer, A.J.; Angyal, A.; et al. The Impact of Viral Mutations on Recognition by SARS-CoV-2 Specific T Cells. *iScience* **2021**, *24*, 103353. https://doi.org/10.1016/j.isci.2021.103353.
97. Chang, C.X.L.; Tan, A.T.; Or, M.Y.; Toh, K.Y.; Lim, P.Y.; Chia, A.S.E.; Froesig, T.M.; Nadua, K.D.; Oh, H.-L.J.; Leong, H.N.; et al. Conditional Ligands for Asian HLA Variants Facilitate the Definition of CD8+ T-Cell Responses in Acute and Chronic Viral Diseases. *Eur. J. Immunol.* **2013**, *43*, 1109–1120. https://doi.org/10.1002/eji.201243088.
98. Ma, T.; Ryu, H.; McGregor, M.; Babcock, B.; Neidleman, J.; Xie, G.; George, A.F.; Frouard, J.; Murray, V.; Gill, G.; et al. Protracted yet Coordinated Differentiation of Long-Lived SARS-CoV-2-Specific CD8+ T Cells during Convalescence. *J. Immunol.* **2021**, *207*, 1344–1356. https://doi.org/10.4049/jimmunol.2100465.
99. Harndahl, M.; Lamberth, K.; Roder, G.; Justesen, S.; Madsen, M.; Nielsen, M.; Lundegaard, C.; Larsen, M.V.; Tang, S.; Brunak, S.; et al. Large Scale Analysis of Peptide-HLA Class I Interactions (Direct IEDB Submission 1019519). 2010. Available online: http://www.iedb.org/reference/1019519 (accessed on 7 March 2020).
100. Gupta, V.; Tabiin, T.M.; Sun, K.; Chandrasekaran, A.; Anwar, A.; Yang, K.; Chikhlikar, P.; Salmon, J.; Brusic, V.; Marques, E.T.A.; et al. SARS Coronavirus Nucleocapsid Immunodominant T-Cell Epitope Cluster Is Common to Both Exogenous Recombinant and Endogenous DNA-Encoded Immunogens. *Virology* **2006**, *347*, 127–139. https://doi.org/10.1016/j.virol.2005.11.042.
101. Yang, K.; Sun, K.; Srinivasan, K.N.; Salmon, J.; Marques, E.T.; Xu, J.; August, J.T. Immune Responses to T-Cell Epitopes of SARS CoV-N Protein Are Enhanced by N Immunization with a Chimera of Lysosome-Associated Membrane Protein. *Gene Ther.* **2009**, *16*, 1353–1362. https://doi.org/10.1038/gt.2009.92.

102. Zhang, J.; Lu, D.; Li, M.; Liu, M.; Yao, S.; Zhan, J.; Liu, W.J.; Gao, G.F. A COVID-19 T-Cell Response Detection Method Based on a Newly Identified Human CD8+ T Cell Epitope from SARS-CoV-2—Hubei Province, China, 2021. *China CDC Wkly.* **2022**, *4*, 83–87. https://doi.org/10.46234/ccdcw2021.258.
103. Sylvester-Hvid, C.; Nielsen, M.; Lamberth, K.; Røder, G.; Justesen, S.; Lundegaard, C.; Worning, P.; Thomadsen, H.; Lund, O.; Brunak, S.; et al. SARS CTL Vaccine Candidates; HLA Supertype-, Genome-Wide Scanning and Biochemical Validation. *Tissue Antigens* **2004**, *63*, 395–400. https://doi.org/10.1111/j.0001-2815.2004.00221.x.
104. Rasmussen, M.; Harndahl, M.; Kristensen, A.B.; Nielsen, I.K.; Jorgensen, K.W.; Stryhn, A.; Nielsen, M.; Buus, S. Large Scale Analysis of Peptide—HLA-I Stability (Direct IEDB Submissions 1028282, 1028285, 1028287, 1028288, 1028289, 1028290, 1028291, 1028292, 1028293). 2014. Available online: http://www.iedb.org/reference/1028282 (accessed on 23 April 2020).
105. He, Y.; Zhou, Y.; Wu, H.; Kou, Z.; Liu, S.; Jiang, S. Mapping of Antigenic Sites on the Nucleocapsid Protein of the Severe Acute Respiratory Syndrome Coronavirus. *J. Clin. Microbiol.* **2004**, *42*, 5309–5314. https://doi.org/10.1128/JCM.42.11.5309-5314.2004.
106. Arya, R.; Kumari, S.; Pandey, B.; Mistry, H.; Bihani, S.C.; Das, A.; Prashar, V.; Gupta, G.D.; Panicker, L.; Kumar, M. Structural Insights into SARS-CoV-2 Proteins. *J. Mol. Biol.* **2021**, *433*, 166725. https://doi.org/10.1016/j.jmb.2020.11.024.
107. Liu, J.; Sun, Y.; Qi, J.; Chu, F.; Wu, H.; Gao, F.; Li, T.; Yan, J.; Gao, G.F. The Membrane Protein of Severe Acute Respiratory Syndrome Coronavirus Acts as a Dominant Immunogen Revealed by a Clustering Region of Novel Functionally and Structurally Defined Cytotoxic T-Lymphocyte Epitopes. *J. Infect. Dis.* **2010**, *202*, 1171–1180. https://doi.org/10.1086/656315.
